# A novel focal adhesion-related risk model predicts prognosis of bladder cancer —— a bioinformatic study based on TCGA and GEO database

**DOI:** 10.1186/s12885-022-10264-5

**Published:** 2022-11-10

**Authors:** Jiyuan Hu, Linhui Wang, Luanfeng Li, Yutao Wang, Jianbin Bi

**Affiliations:** 1 Department of Urology, The First Affiliated Hospital of China Medical University, Shenyang, Liaoning China; 2 Department of Pharmacology, China Medical University, School of Pharmacy of China Medical University, Shenyang, Liaoning China

**Keywords:** Bladder cancer, Computational biology, Risk signature, Immune infiltration, Focal adhesion

## Abstract

**Background:**

Bladder cancer (BLCA) is the ninth most common cancer globally, as well as the fourth most common cancer in men, with an incidence of 7%. However, few effective prognostic biomarkers or models of BLCA are available at present.

**Methods:**

The prognostic genes of BLCA were screened from one cohort of The Cancer Genome Atlas (TCGA) database through univariate Cox regression analysis and functionally annotated by Kyoto Encyclopedia of Genes and Genomes (KEGG) pathway enrichment analysis. The intersecting genes of the BLCA gene set and focal adhesion-related gene were obtained and subjected to the least absolute shrinkage and selection operator regression (LASSO) to construct a prognostic model. Gene set enrichment analysis (GSEA) of high- and low-risk patients was performed to explore further the biological process related to focal adhesion genes. Univariate and multivariate Cox analysis, receiver operating characteristic (ROC) curve analysis, and Kaplan–Meier survival analysis (KM) were used to evaluate the prognostic model. DNA methylation analysis was presented to explore the relationship between prognosis and gene methylation. Furthermore, immune cell infiltration was assessed by CIBERSORT, ESTIMATE, and TIMER. The model was verified in an external GSE32894 cohort of the Gene Expression Omnibus (GEO) database, and the Prognoscan database presented further validation of genes. The HPA database validated the related protein level, and functional experiments verified significant risk factors in the model.

**Results:**

VCL, COL6A1, RAC3, PDGFD, JUN, LAMA2, and ITGB6 were used to construct a prognostic model in the TCGA-BLCA cohort and validated in the GSE32894 cohort. The 7-gene model successfully stratified the patients into both cohorts’ high- and low-risk groups. The higher risk score was associated with a worse prognosis.

**Conclusions:**

The 7-gene prognostic model can classify BLCA patients into high- and low-risk groups based on the risk score and predict the overall survival, which may aid clinical decision-making.

**Supplementary Information:**

The online version contains supplementary material available at 10.1186/s12885-022-10264-5.

## Background

According to Cancer Statistics 2021, published by American Cancer Society, bladder cancer (BLCA) is the ninth most commonly diagnosed cancer globally and is the fourth most common malignancy in men [1]. Over 570,000 new cases of BLCA and 210,000 deaths were recorded in 2020 alone, indicating poor prognosis [2]. Men are at four times the risk of developing BLCA than women [3]. The significant risk factors of BLCA are advanced age (between 70 and 84 years) and cigarette smoking. In fact, approximately 50% of BLCA patients are smokers [4]. Furthermore, almost 3/4^th^ of the diagnosed cases are non-muscular invasive bladder cancer (NMIBC), often treated with transurethral resection of bladder tumors (TRUBT) and intravesical therapy. Muscular invasive bladder cancer (MIBC) is relatively rare and is generally treated by radical cystectomy and neoadjuvant chemotherapy [5, 6]. Although pathological biopsies and cystoscopies are routinely used to detect BLCA, these methods are invasive and inconvenient. Although several urine biomarkers of BLCA have been confirmed by the US Food and Drug Administration (FDA), they lack the diagnostic accuracy to replace cystoscopy [4]. Therefore, this study aimed to identify novel, effective diagnostic biomarkers of BLCA.

Focal adhesion (FA) is a group of macromolecular proteins that connect the ends of specialized actin fibers to the extracellular matrix (ECM) and enable cell migration, which is critical to the process of tumor metastasis [7]. FAs are frequently downregulated during cancer metastasis, although some FA components are upregulated in some invasive tumors [8]. Thus, FAs are increasingly being considered therapeutic targets of cancer.

Analysis of gene expression datasets from The Cancer Genome Atlas (TCGA) and Gene Expression Omnibus (GEO) have helped identify prognostic gene signatures of various cancers. For instance, a predictive model consisting of hypoxia gene signatures was constructed for BLCA based on TCGA and GEO databases [9]. In addition, a risk score model of epithelial-mesenchymal transition (EMT)-related gene signature was also developed to predict BLCA prognosis based on the two databases [10]. A recent study established an 11-gene prognostic signature of BLCA based on five cohorts from TGCA and GEO [11]. However, the prognostic value of FAs has not been ascertained by any study so far. Therefore, this study aimed to explore the relationship between FAs and BLCA prognosis using the bioinformatics approach and establish a predictive model based on the risk score.

## Materials and methods

### Data source and processing

TCGA and GEO databases were screened for BLCA cohorts with a sample size > 150, including clinical data such as overall survival duration, survival status, gender, age, histological grade, pathological stage, TNM stage, and lymphatic stage metastasis. The GEO cohorts were further screened based on additional requirements for the verification set. The gene expression matrix dataset (HTSeq-FPKM) of bladder cancer (*n* = 430) was downloaded from TCGA on UCSC Xena, and the clinical data were obtained from cBioPortal (http://www.cbioportal.org). The external GSE32894 cohort (*n* = 308) with expression matrix and clinical data [12] was acquired from the GEO database.

### Selection of FA-related differentially expressed genes (DEGs)

TCGA-BLCA cohort was set as the training set, and prognostically relevant genes were screened using the univariate Cox analysis with a *p*-value < 0.01 as the criterion. R software package “limma” was used to identify DEGs between the BLCA and normal bladder samples in the same cohort from these selected genes [13]. The threshold was set as |log (fold change) |> 1, and the adjusted *p*-value < 0.01. The significant DEGs related to BLCA prognosis intersected with 199 FA-related genes obtained from the Molecular Signatures Database (MSigDB) of GSEA using keywords KEGG_FOCAL_ADHESION [14] using a Venn diagram.

### Establishment of the predicting model related to risk score

A prognostic model was constructed with the intersecting genes identified as above by LASSO regression using the R software packages “glmnet” [15] and “survival”. The “CV.glmnet” function can randomly simulate 1000 times for k-fold cross-validation (k = 10). The dataset was automatically divided into 10 equal portions in the tenfold cross-validation. One random part was selected as the validation set, and the remaining 9 parts as training sets. The deviance of the 10 tests was used to evaluate the accuracy of the tenfold CV, and minimum deviance indicated the best performance of the model. The regression coefficients of individual genes were determined, and genes with regression coefficient approaching 0 with the increase in Lambda were excluded. The remaining candidate genes were used to construct the model, and the risk score of each patient in the TGCA-BLCA cohort was calculated as ∑${7}_{i}$(${m}_{i}$ · ${n}_{i}$), where 7 is the number of candidate genes, ${m}_{i}$ is the gene expression value and ${n}_{i}$ is regression coefficient.

### KEGG enrichment analysis

The latest KEGG Pathway gene annotations were obtained through KEGG rest API ( https://www.kegg.jp/kegg/rest/keggapi.html) in the KEGG official database [16]. KEGG pathway enrichment analysis was then performed using the R software package “clusterProfiler” through an online tool called Sangerbox (http://www.sangerbox.com/tool). The threshold for statistical significance were *p* < 0.05 and FDR of < 0.1.

### Gene set enrichment analysis (GSEA)

GSEA software and predefined gene set files were downloaded from https://www.gsea-msigdb.org, and the samples were divided into high- and low-risk groups based on the risk score. The number of permutations was set as 1000.

### Univariate and multivariate Cox regression analyses

The R software package "survival" was used for univariate and multivariate Cox regression analysis of the risk score and clinicopathological factors, including age, gender, pathological stage, T stage, histological grade, lymphatic metastasis, and angio-lymphatic invasion. Only the statistically significant factors in the univariate Cox analysis were included in the multivariate Cox model.

### ROC analysis

The R software package “pROC” was used for receiver operating characteristic (ROC) analysis. The area under the curve (AUC) was obtained, and the confidence interval was evaluated. The cut-off values for 1-, 3- and 5-year overall survival (OS) were calculated.

### Kaplan–Meier analysis

The R software package “Survival” was used to integrate the OS rate and duration with the gene expression data of both TCGA-BLCA and GSE32894 cohorts. The prognostic significance of each gene was evaluated by the Cox method. The patients were divided into the high- and low-risk groups using the cut-off value of 3-year OS. For the subgroups based on clinical variables and the expression levels of the 7 candidate genes, the best cut-off value for the risk score was calculated using the R software package “Maxstat”. The minimum sample size was set at > 25%, and the maximum sample size at < 75%, and the patients were divided into high- and low-risk groups.

### DNA methylation analysis

It is believed that DNA methylation is responsible for influencing prognosis in cancer development. An online tool MethSurv (https://biit.cs.ut.ee/methsurv/) was used to explore the prognostic patterns of single CpG methylation of the 7 genes in bladder cancer [17]. Only the most significant prognostic *p*-values were selected (likelihood ratio (LR) test *p*-value).

### Immune environment evaluation

The R packages “CIBERSORT” (used to calculate the cell composition as a function of gene expression profile) and “ESTIMATE” (used to calculate the fraction of stromal and immune cells according to gene expression level) were used to calculate the number of infiltrating immune cells, immune score, stromal score and tumor purity in each patient from TCGA-BLCA cohort [18, 19]. Twenty-two immune cell genotypes were obtained by combining CIBERSORT with LM22, a gene matrix downloaded from the CIBERSORT website (https://cibersort.stanford.edu/), within the R software. The differences between the risk groups were analyzed, and the immune score and risk score were combined for survival analysis. Besides, the TIMER platform (http://timer.cistrome.org/) was also used to verify the immune infiltration analysis completed by CIBERSORT. The “gene module” of immune association was presented to evaluate the correlation between immune cells and every 7 genes in the prognostic model [20].

### Verification of the prognostic model

The accuracy of the prognostic model was tested on the external GSE32894 dataset. Besides, the Prognoscan database (www.prognoscan.org) was also applied to validate further the correlation between gene expression and overall survival time [21], where GSE5287 and GSE13507 were utilized. The protein expression of individual genes in the model in cancer and normal tissues was also observed in the Human Protein Atlas (HPA) database (http://www.proteinatlas.org/), so as further to validate the genes in our model [22].

### Cell culture and small interfering RNA (siRNA) transfection

The human BLCA cell line T24 was used in this study, purchased from the Chinese Academy of Sciences cell bank. T24 was cultured in RPMI-1640 medium (Procell) with 10% fetal bovine serum. The sequence of siRNA targeting COL6A1 and LAMA2 purchased from JTSBIO Co., were listed in Supplementary Table 5.

### RNA extraction and quantitative real-time PCR (qRT-PCR)

Extraction of RNA was performed with RNAiso Plus (Takara). Prime Script RT Master Mix (Takara) was used to reverse transcription then cDNA was produced. The SYBR kit (Takara) was used to perform qRT-PCR. The relative expression of the gene was calculated by the 2^−ΔΔCt^ method. The primer sequences targeting COL6A1, LAMA2 and GAPDH were listed in Supplementary Table 6.

### Wound-healing assay

The BLCA cells were seeded in a six-well plate. When the density reached more than 90%, a straight line was drawn with a 200-ul tip. Cultivation of cells was continued with a low-serum medium containing 3% serum. Photographs were taken at 0 h and 48 h, and then the speed of scratch healing was compared between the different groups.

### Transwell assay

Six hundred ul of medium containing 10% serum was added to the lower chamber of a 24-well plate. Each 200 ul BLCA cell suspension was inoculated in the upper chamber. Transwell chambers with 8-μm-pore were used for cell migration assay. Following incubation for 24 h, cells beneath the membrane were stained with crystal violet, and cells above the membrane were washed off and imaged by microscopy.

### Statistical analysis

All the statistical analysis was completed by software R. The Logrank test was used to assess the significance of prognostic differences between different groups in the Kaplan–Meier analysis. The Kruskal–Wallis rank sum test was used in multiple groups comparisons of clinical sub-group analysis. Univariate analysis and multivariate analysis were performed using Cox regression analysis with the R package “survival”. The R package “limma” was used to identify DEGs between the tumor and normal samples in the same TCGA-BLCA cohort from these selected genes. The R package “glmnet” was used in LASSO regression to establish the predicting model. A *p*-value < 0.05 was considered as statistically significant.

## Results

### Data extraction

**Fig. 1:**
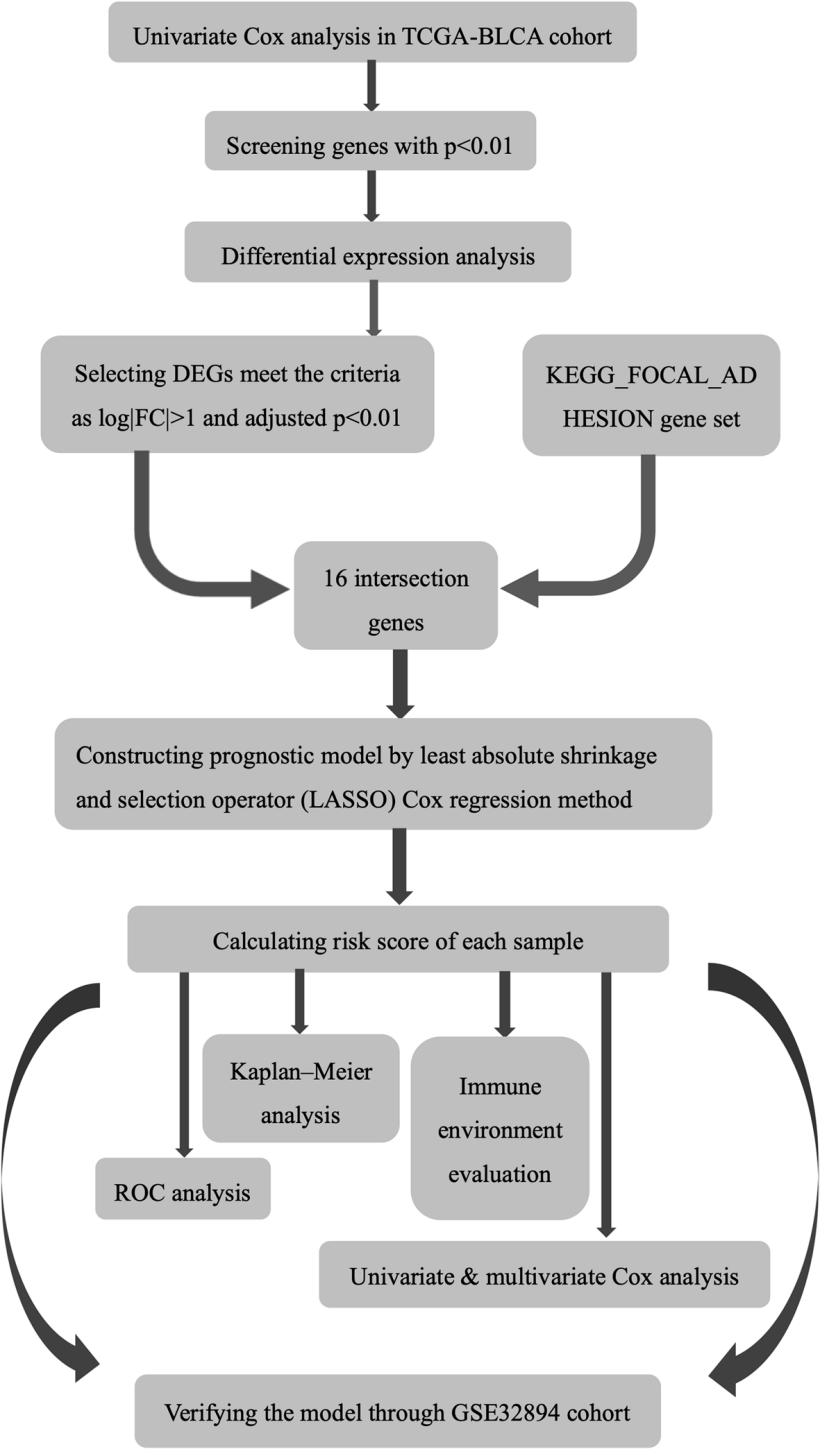
Flow chart of the study process

The gene expression and clinical data of BLCA samples were retrieved from TCGA (*n* = 430) and GSE32894 (*n* = 308). After filtering the data, there were 403 cases in the TCGA-BLCA cohort and 224 cases in the GSE32894 cohort. The flow chart is shown in Fig. 1. Clinical data regarding age, gender, histological grade, WHO grade, pathological stage, T stage, lymphatic node metastasis, and angiolymphatic invasion of the two cohorts are summarized in Table 1.

**Table 1:** Clinical information table. The major clinical factors related to prognosis were listed as following

Clinical factors	TCGA-BLCA *n* = 403%		GSE32894 *n* = 224%	
**Age**				
> 60	296	73.45	178	79.46
≤ 60	107	26.55	46	20.54
**Gender**				
Male	298	73,95	163	72.77
Female	105	26.05	61	27.23
**Pathological stage**				
Stage I&II	130	32.26	-	-
Stage III	140	34.74	-	-
Stage IV	131	32.51	-	-
unknown	2	0.49	-	-
**T stage**				
≤ T2	121	30.02	216	96.43
T3	191	47.39	7	1.74
T4	57	14.14	1	0.25
unknown	34	8.44	-	-
**Lymphatic node metastasis**				
Negative	235	58.31	-	-
Positive	125	31.02	-	-
Unknown	43	10.67	-	-
**Angiolymphatic Invasion**				
Negative	127	31.52	-	-
Positive	148	36.72	-	-
unknown	128	31.76	-	-
**Histological grade**				
High	380	94.53	-	-
Low	20	4.98	-	-
**Vital status**				
Alive	225	55.83	199	88.84
Dead	178	45.17	25	11.16
**Grade (WHO1999)**				
G1	-	-	45	20.09
G2	-	-	84	37.50
G3	-	-	93	41.52

### Establishment of a prognostic model based on the risk score

**Fig. 2:**
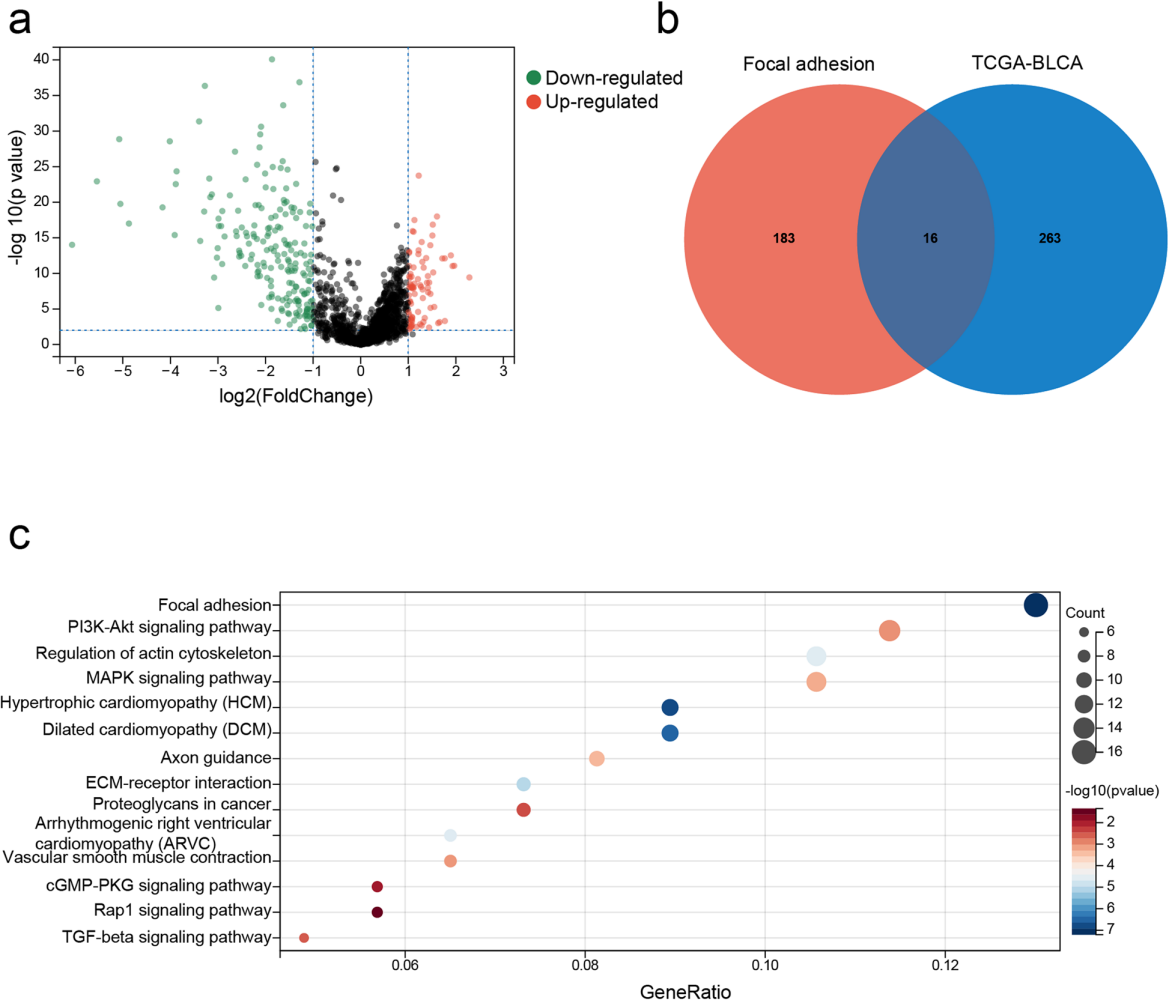
Identification of Focal Adhesion (FA)-related prognostic genes. **a** Volcano plot showing the differentially expressed genes (DEGs) between the tumor and normal tissue samples in the TCGA-BLCA cohort. The red dots indicate a higher expression of genes in tumor tissues, and the green dots indicate a lower expression of genes in tumor tissues. **b** Venn diagram showing the intersection of FA-related genes and BLCA DEGs. **c** Bubble plot showing the enriched KEGG pathways of DEGs (www.kegg.jp/kegg/kegg1.html). The circle size indicates gene ratio, and the color refers to the *p*-value

**Fig. 3:**
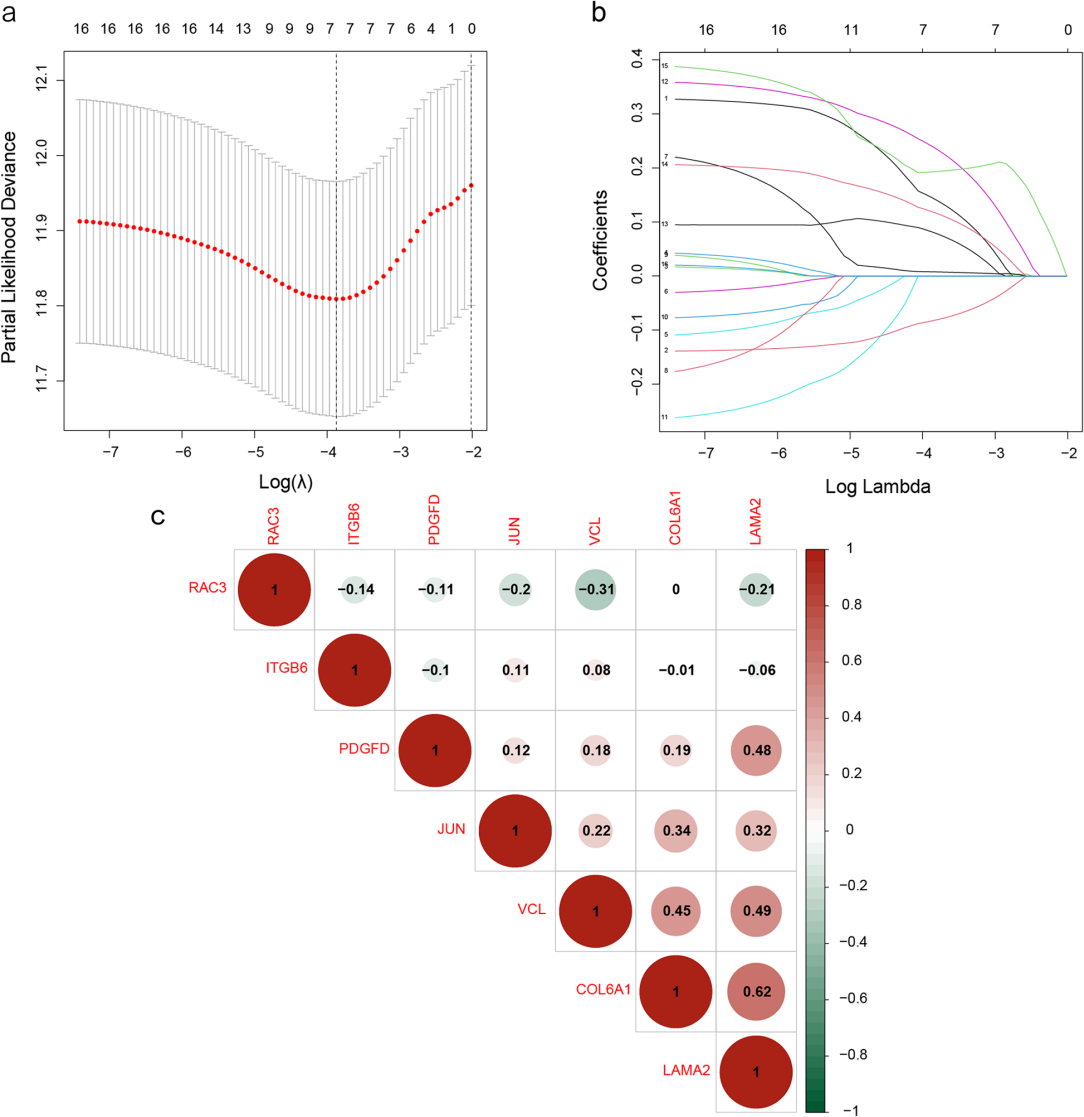
LASSO Cox regression analysis. **a** Line graph of regression coefficients and Lambda value. Different colors represented different genes. The regression coefficients of some genes dropped to 0 with an increase in Lambda, indicating that they may not affect the model. **b** The deviance of the cross-validation. Minimum deviance indicates optimal model performance. **c** The co-expression heat map of 11 genes. Red indicates positive correlation, and green indicates negative correlation

Univariate Cox analysis of the TCGA-BLCA cohort identified 2461 genes (*p* < 0.01), of which 274 were differentially expressed between the tumor and non-tumor samples (Fig. 2a). Sixteen DEGs intersected with FA-related genes (Fig. 2b), and were functionally annotated by KEGG pathway enrichment analysis (Fig. 2c). The detailed information of these genes is listed in Supplementary Table 1. The above DEGs were subjected to Lasso regression, and 7 genes with the smallest deviance were included in the prognostic model (Figs. 3a, b). The coefficient values and other details of these genes are listed in Supplementary Table 2, and outcomes of univariate regression analysis are summarized in Supplementary Table 3. The risk score was calculated as *VCL* * 0.1452—*ITGB6* * 0.0832 + *COL6A1* * 0.0077 + *RAC3* * 0.2404 + *PDGFD* * 0.0817 + *JUN* * 0.1192 + *LAMA2* * 0.1927. Furthermore, we performed a matrix correlation analysis to determine any collinear relationship between these genes. As shown in Fig. 3c, apart from COL6A1 and LAMA2, the co-expression indices of the other gene pairs were all < 0.5.

### Gene set enrichment analysis (GSEA)

**Fig. 4:**
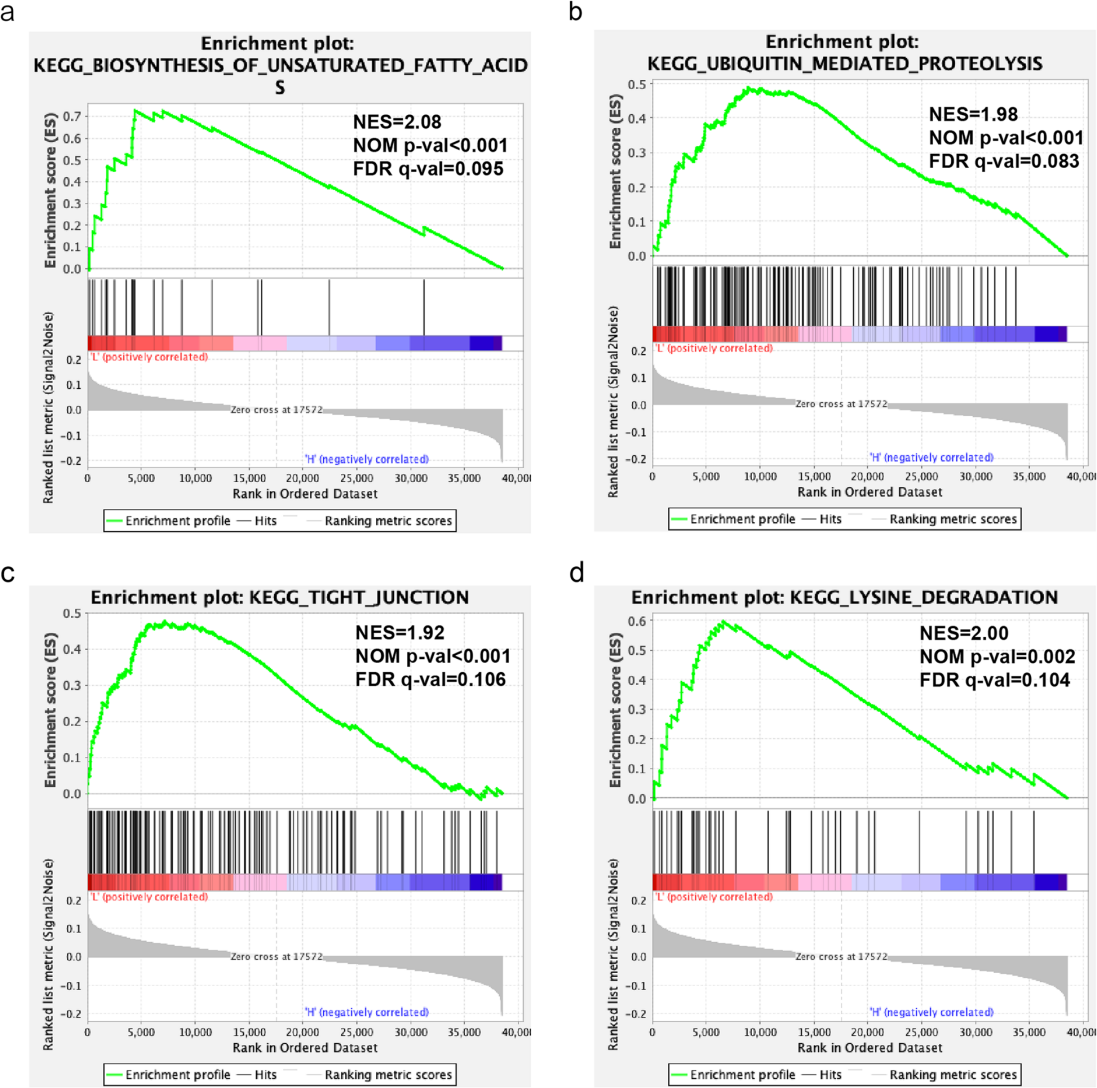
Gene set enrichment analysis (GSEA) of high and low-risk score groups. **a** Enrichment plot of KEGG_BIOSYNTHESIS_OF_UNSATURATED_FATTY_ACIDS. **b** Enrichment plot of KEGG_UBIQUITIN_MEDIATED_PROTEOLYSIS. **c** Enrichment plot of KEGG_TIGHT_JUNCTION. (d) Enrichment plot of KEGG_LYSINE_DEGRADATION

The 403 samples in the TCGA-BLCA cohort were stratified into high- and low-risk groups based on the 7-gene risk score. GSEA further indicated that the high-risk group was significantly associated with biosynthesis of unsaturated fatty acids, tight junction, lysine degradation, and ubiquitin-mediated proteolysis (*p* < 0.005; Fig. 4).

### Kaplan–Meier analysis of 7 genes

**Fig. 5:**
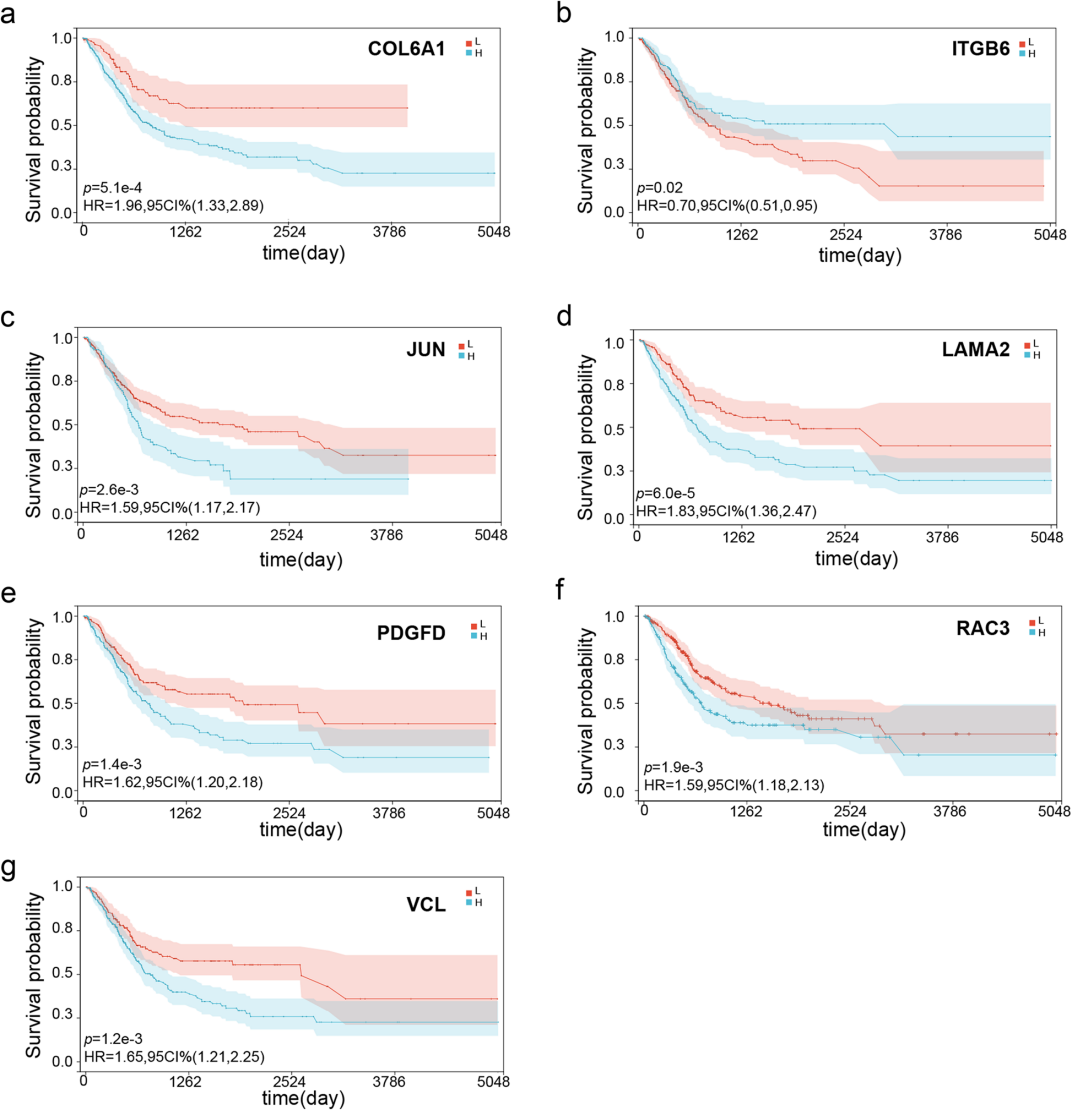
Kaplan–Meier (KM) survival analysis of patients divided into the high- and low-expression groups of the 7 prognostic genes in the TCGA-BLCA cohort (training set). Blue represents high-risk group, red represents low-risk group. **a** COL6A1 **b** ITGB6 **c** JUN **d** LAMA2 **e** PDGFD **f** RAC3 **g** VCL

**Fig. 6:**
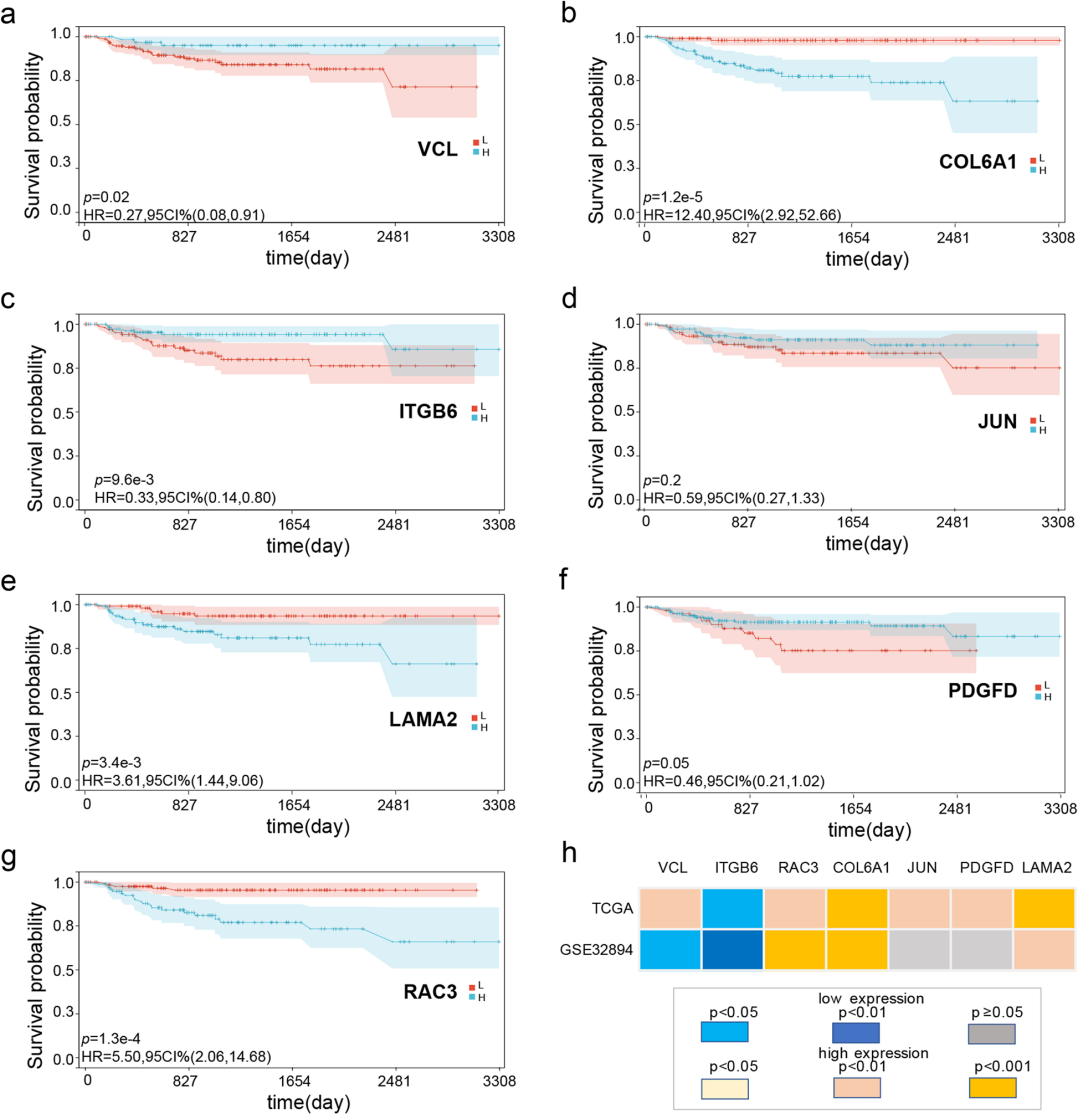
Kaplan–Meier (KM) survival analysis of patients divided into the high- and low-expression groups of the 7 prognostic genes in the GSE32894 cohort (test set). Blue represents high-risk group, red represents low-risk group. **a** VCL **b** COL6A1 **c** ITGB6 **d** JUN **e** LAMA2 **f** PDGFD **g** RAC3 **h** Heatmap of gene expression level and prognosis. Yellow indicates that high gene expression leads to worse prognosis, blue indicates that low gene expression leads to worse prognosis, and grey indicates a lack of significance (*p* > 0.05). The deeper color refers to smaller *p*-value

The patients in the TCGA-BLCA cohort were stratified into high- and low-expression subgroups for each of the 7 prognostic genes and subjected to Kaplan–Meier analysis to determine their impact on survival. These seven genes include COL6A1 (Fig. 5a), ITGB6 (Fig. 5b), JUN (Fig. 5c), LAMA2 (Fig. 5d), PDGFD (Fig. 5e), RAC3 (Fig. 5f) and VCL (Fig. 5g). And the GSE32894 cohort was divided with the same criterion for validation. These seven genes include VCL (Fig. 6a), COL6A1 (Fig. 6b), ITGB6 (Fig. 6c), JUN (Fig. 6d), LAMA2 (Fig. 6e), PDGFD (Fig. 6f) and RAC3 (Fig. 6g). The heatmap demonstrated correlation between gene and survival (Fig. 6h), except for JUN and PDGFD in GSE32894; all genes comprising the prognostic model were significantly associated with the survival of BLCA patients (*p* < 0.05). The high expression of ITGB6 indicated a better prognosis in both the training and validation sets. In contrast, increased expression of RAC3, COL6A1, and LAMA2 was correlated with worse prognosis in both sets. Interestingly, high expression of VCL was associated with a worse prognosis in the training set but indicated a favorable prognosis in the validation set. Taken together, ITGB6, RAC3, COL6A1, and LAMA2 could accurately predict patient prognosis.

### Univariate and multivariate Cox regression analysis

**Fig. 7:**
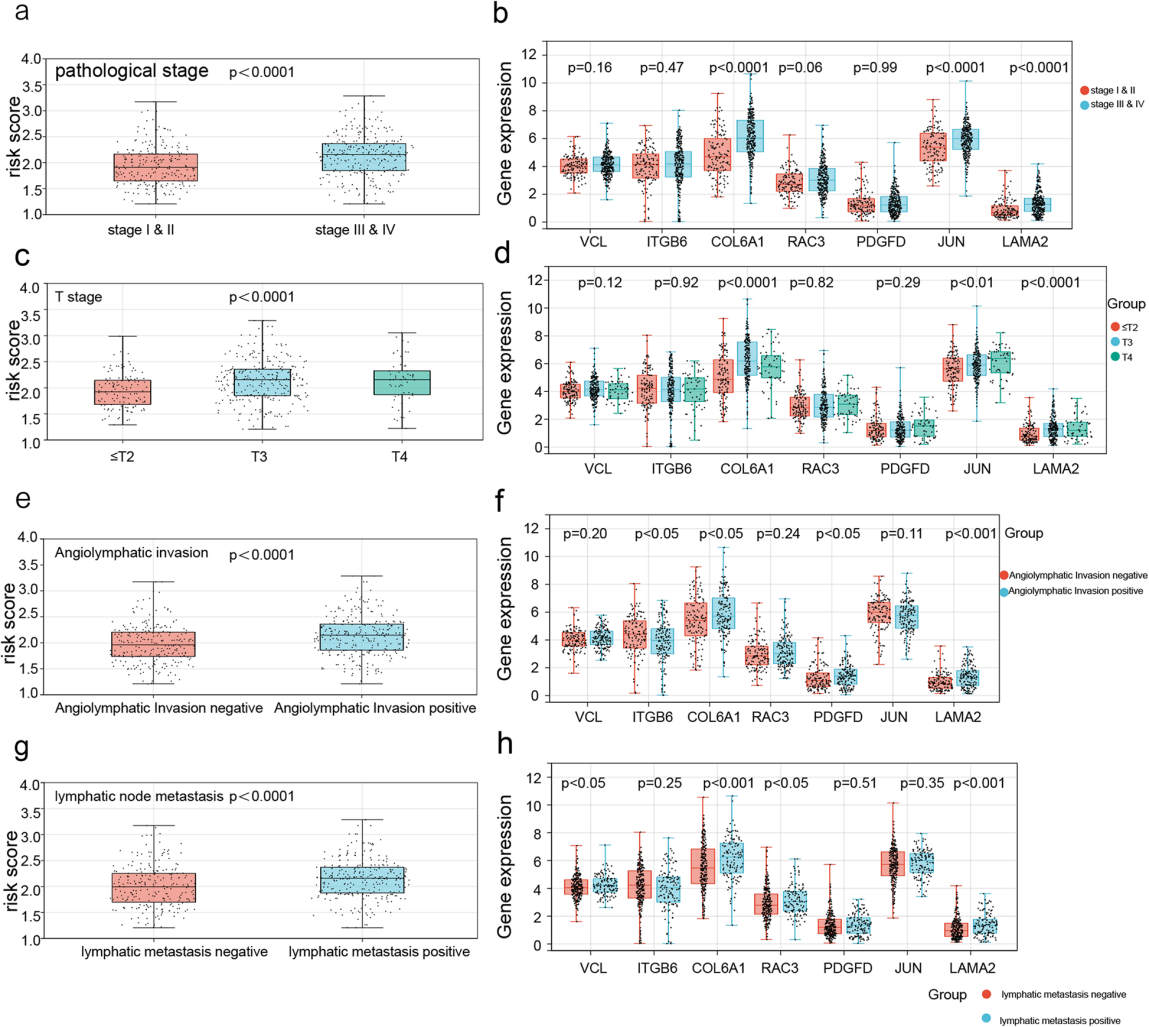
Risk scores and expression levels of the 7 prognostic genes in the different clinical sub-groups of TCGA-BLCA cohort. Risk scores in subgroups of **a** pathological stages, **c** T stages, **e** angiolymphatic invasion, and **g** lymphatic node metastasis. Gene expression levels in subgroups of **b** pathological stages, **d** T stages, **f** angiolymphatic invasion, and **h** lymphatic node metastasis

**Fig. 8:**
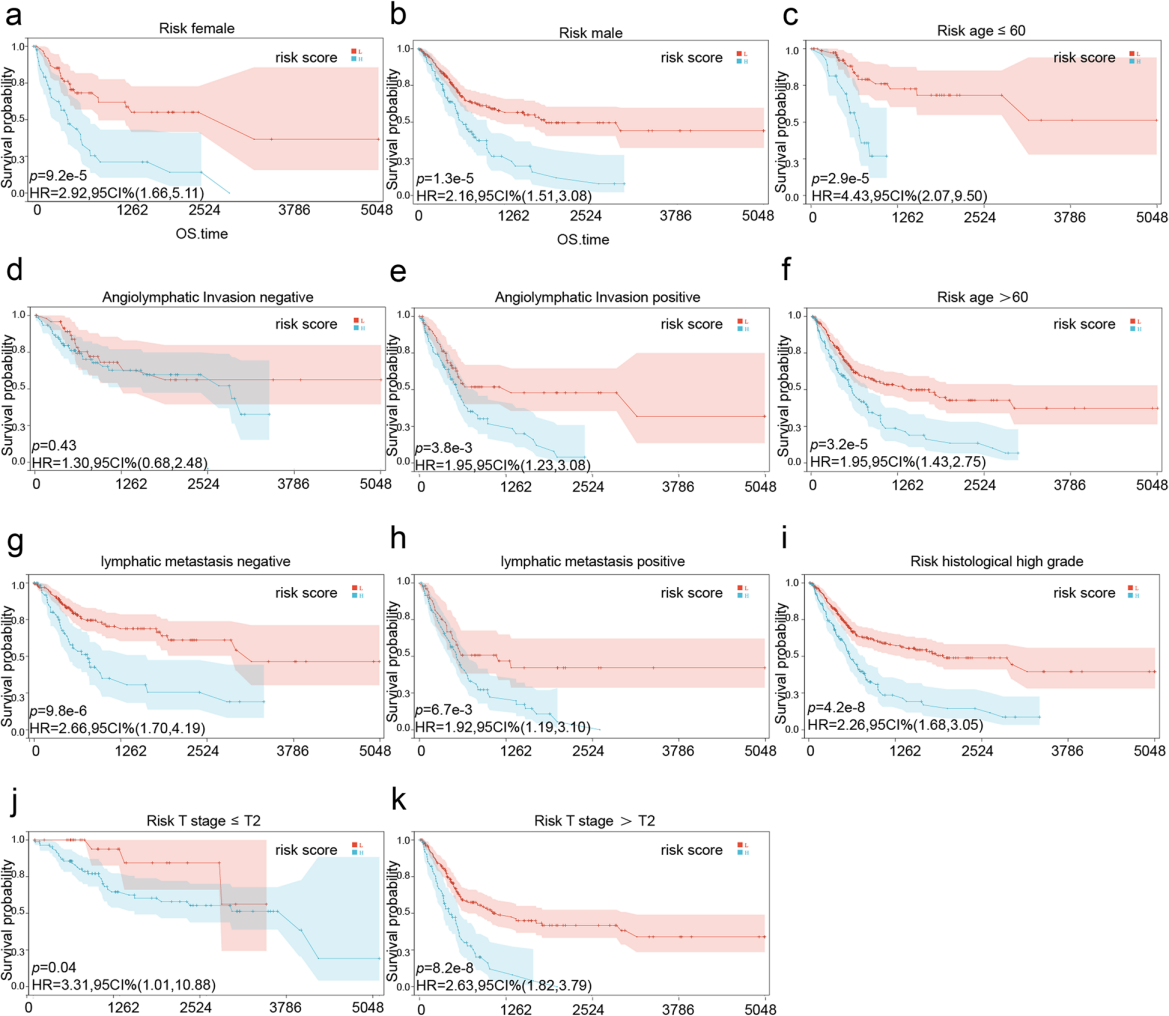
Kaplan–Meier survival curves of high- and low-risk groups stratified based on **a-b** gender, **c-d** angiolymphatic invasion, **e–f** lymphatic node metastasis, **g-h** T stage, **i-j** age, and **k** histological grade. Blue high- represents risk group, red represents low-risk group

Univariate and multivariate Cox regression analyses identified age, angiolymphatic invasion, and the risk score as independent prognostic factors of BLCA (Table 2). Furthermore, ROC curve analysis of the three factors indicated that the AUC of the risk score was greater than that of the other two factors (Supplementary Fig. 1), suggesting more substantial predictive accuracy. Furthermore, higher risk scores correlated with more advanced angiolymphatic invasion, T stage, pathological stage, and lymphatic node metastasis. The patients were divided into subgroups based on these clinical factors, and the expression levels of the 7 prognostic genes were compared. As shown in Fig. 7, the expression levels of COL6A1 and LAMA2 were significantly different across all subgroups. Furthermore, the high- and low-risk groups had very different survival rates in subgroups demarcated by age, gender, histological grade, lymphatic node metastasis, and T stage (Fig. 8), indicating that the risk score can predict the prognosis in clinical sub-groups and may assist in clinical decision making.

**Table 2:** Univariate and multivariate Cox regression analysis of clinical factors. (HR: hazard ratio; CI: confidence interval)

	HR (95%CI)	*P* value	HR (95%CI)	*P* value
TCGA				
Age	1.035 (1.019 ~ 1.051)	1.82e-05*	1.039 (1.018 ~ 1.061)	< 0.001*
Gender-male	0.872 (0.630 ~ 1.206)	0.407		
Pathological Stage	1.703 (1.405 ~ 2.064)	5.63e-08*	1.236 (0.615 ~ 2.485)	0.552
T stage	1.702 (1.353 ~ 2.142)	5.79e-06*	1.173 (0.806 ~ 1.706)	0.405
Histological grade	2.915 (0.721 ~ 11.78)	0.133		
Lymphatic metastasis	2.227 (1.625 ~ 3.051)	6.29e-07*	0.967 (0.385 ~ 2.427)	0.942
Angiolymphatic invasion	2.339 (1.601 ~ 3.416)	1.1e-05*	1.711 (1.054 ~ 2.777)	0.030*
Risk score	3.584 (2.455 ~ 5.233)	3.84e-11*	2.521 (1.455 ~ 4.367)	< 0.001*

### DNA methylation analysis of 7 genes

We presented prognostic values of DNA methylation clustering the expression levels of each 7 genes of the prognostic model in the TCGA-BLCA cohort by KM curves (Supplementary Fig. 2). The CpG islands corresponding to the smallest likelihood ratio (LR) test *p*-value were chosen in all 7 genes to ensure the statistical significance. The specific CpG resource of each gene was depicted in the figure. Besides, the relationship between DNA methylation and the prognosis of ITGB6 demonstrated its protective effect, displaying the same trend as it does in the 7-gene prognostic model. prognosis of ITGB6 demonstrated its protective effect, displaying the same trend as in the 7-gene prognostic model.

### Immune cell infiltration

**Fig. 9:**
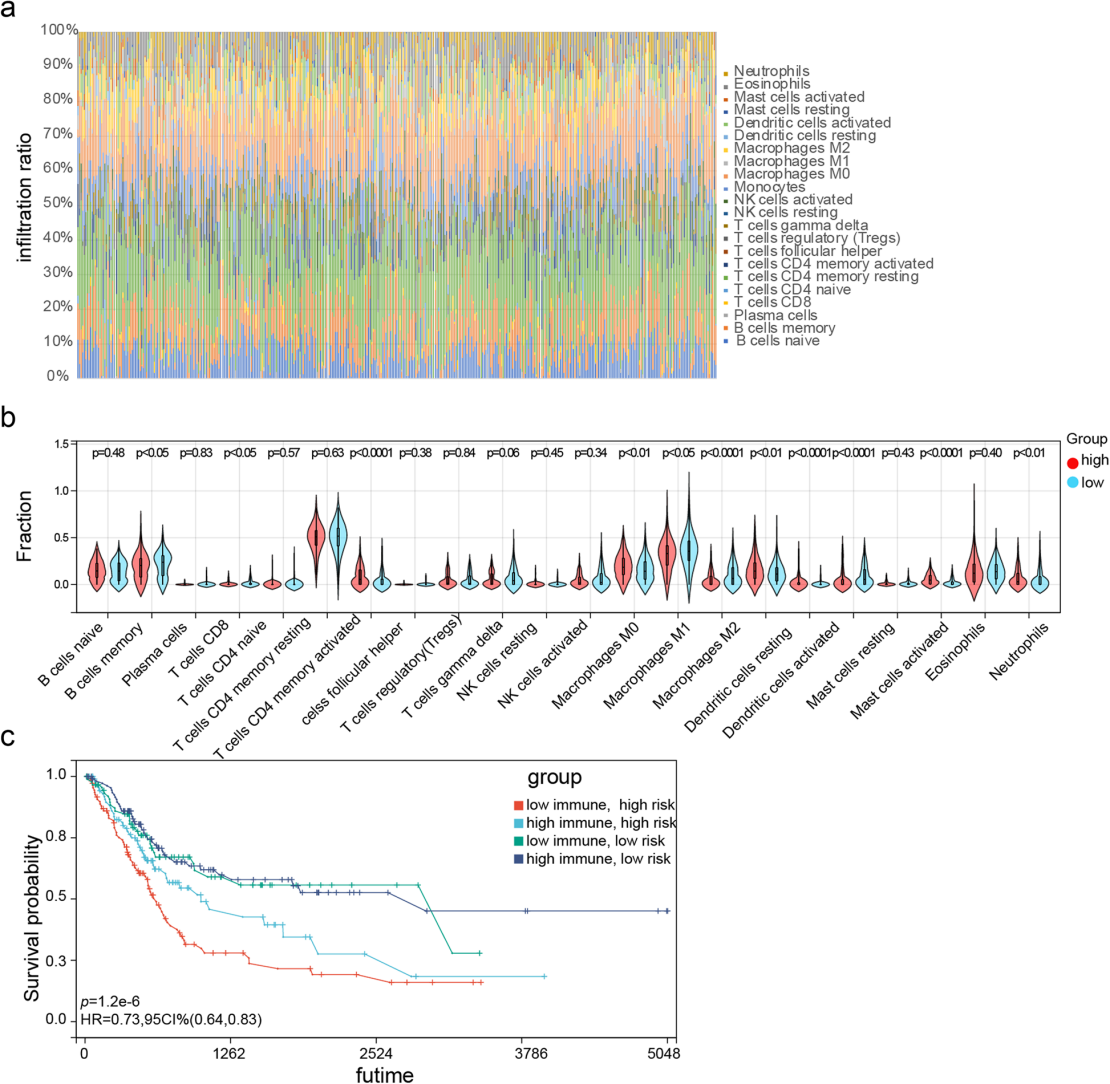
Immune infiltration status. **a** Bar chart showing the infiltration ratio of 22 immune cell types in high and low-risk groups samples. **b** Violin plot showing differences in immune cell types between the high and low-risk groups. **c** KM survival curves of patients stratified by immune score and risk score together

The infiltration ratio of 22 immune cell types was analyzed in the TGCA-BLCA cohort using CIBERSORT (Fig. 9a) and compared between the high- and low-risk groups. As shown in Fig. 9b, the predominant infiltrating immune cells in the high-risk groups were activated CD4 memory T cells, resting dendritic cells (DCs), and activated mast cells, whereas the M1 macrophages and activated DCs showed higher infiltration in the low-risk group (*p* < 0.0001). Kaplan–Meier survival analysis further showed that high infiltration of resting DCs activated mast cells and activated CD4 memory T cells, along with a high-risk score, which portended the worst prognosis. In addition, low infiltration of M1 macrophages and activated DCs in the high-risk group was associated with the worst prognosis (Supplementary Fig. 3). The immune score and tumor purity in TCGA-BLCA cohort were evaluated using the “ESTIMATE” R package (Supplementary Table 4). The patients’ samples were divided into 4 clusters using the median risk and immune scores. As shown in Fig. 9c, patients with the lowest immune score and highest risk score had the worst prognosis. Besides, M0, M2 macrophages, and neutrophils were also statistically significant (*p* < 0.05), thus these immune cells were also selected for further analysis on the TIMER platform. After filtering associated immune cells, M2 macrophages were ultimately chosen to explore further the relationship with the 7 genes (Supplementary Fig. 4). Apart from ITGB6, which showed a negative correlation as a protective factor, the other genes were all positively correlated with Macrophages M2_CIBERSORT as risk factors, demonstrating the same trend as the prognostic model. COL6A1, RAC3, LAMA2, and VCL showed significant statistical meanings (*p* < 0.05).

### Verification of the prognostic model

**Fig. 10:**
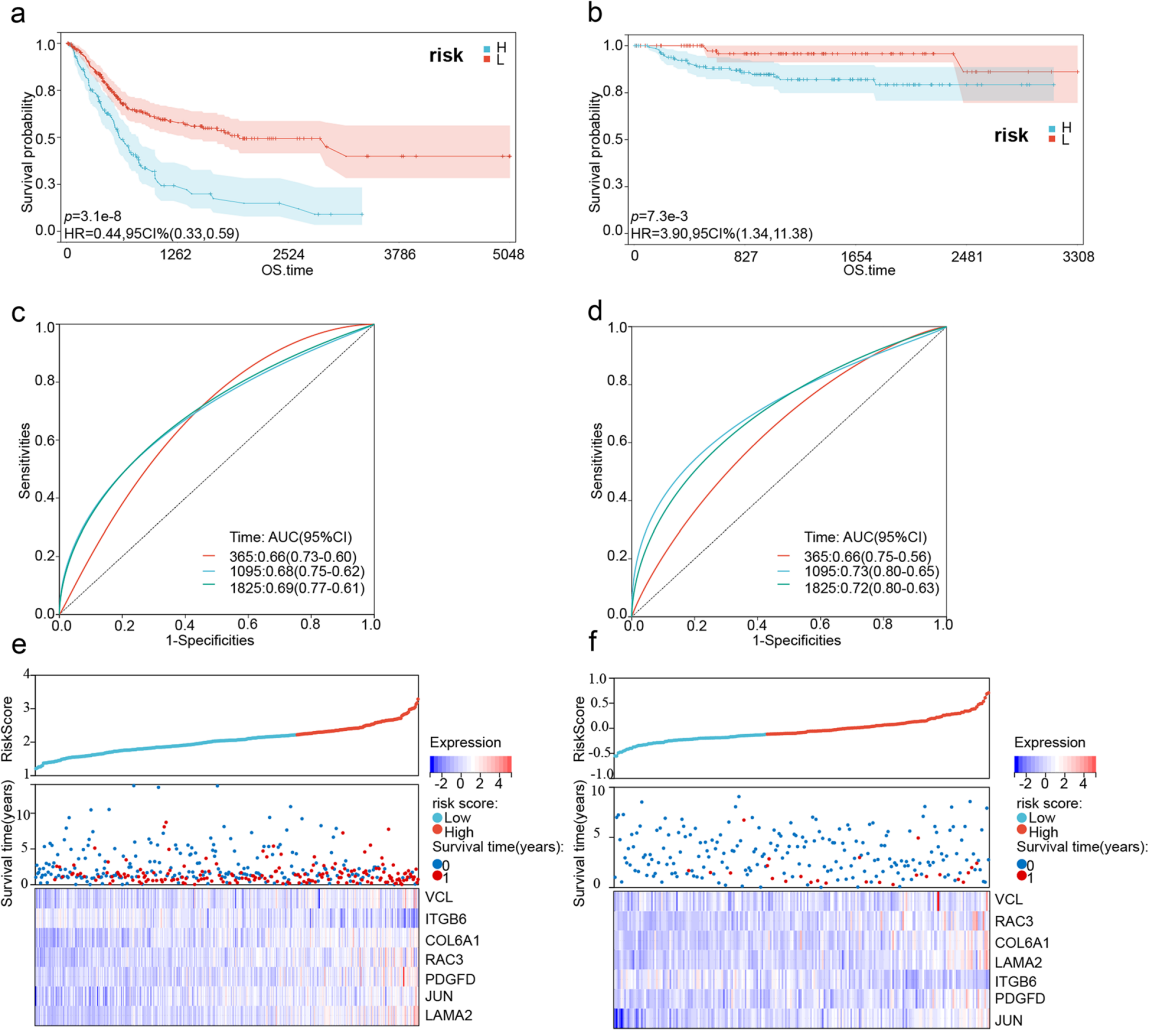
Kaplan–Meier survival curves of the low- and high-risk groups in **a** TCGA-BLCA and **b** GSE32894 cohorts. Blue represents high-risk, red represents low-risk. The ROC curves and corresponding AUC values of the risk score for 1-, 3- and 5-year OS in **c** TCGA-BLCA and **d** GSE32894 cohorts. Risk score distribution, survival status, and gene expression in **e** TCGA-BLCA and **f** GSE32894 cohorts

The optimum cut-off value of the risk score for 3-year OS was -2.2174 and was used to divide the patients in the TCGA-BLCA cohort into the high- and low-risk groups. Likewise, the optimum cut-off value of the validation cohort (GSE32894) was calculated as -0.1290. As shown in Figs. 10a, b, patients in the high-risk group had significantly worse survival than the low-risk group in both the training and verification sets (*p* < 0.05). Furthermore, the AUC values of the risk score for 1-, 3- and 5-year OS were respectively 0.66, 0.68, and 0.69 in the training set, and 0.66, 0.73, and 0.72 in the validation set (Figs. 10c, d). We analyzed the relationship between the risk scores and OS duration and the changes in the expression of various genes in both cohorts (Figs. 10e, f). As expected, ITGB6 was identified as a protective factor in both cohorts. It was downregulated with the increasing risk score. Besides, limited by the sample size of the Prognoscan online tool, RAC3 and COL6A1 were the only risk factors that demonstrated a significant statistical correlation with the overall survival time of patients (Supplementary Figs. 5 and 6, *p* < 0.05) in external dataset GSE13507 and GSE5287, respectively. Though KM curves of ITGB6 in the GSE13507 cohort (Supplementary Fig. 7) still showed a tendency as a protective factor, the *p*-value was > 0.05, with no statistical significance. Taken together, the 7-gene risk model can successfully stratify BLCA patients into prognostic groups. Furthermore, In the validation of protein level on the HPA database, VCL, COL6A1, RAC3, PDGFD and JUN showed higher protein expression in BLCA tissue than in normal tissue. In comparison, ITGB6 showed higher protein expression in normal tissue than in BLCA tissue. In this database, LAMA2 expression was not detected in normal or cancerous tissues (Supplementary Fig. 8).

### COL6A1 and LAMA2 significantly promotes BLCA cell migration

**Fig. 11:**
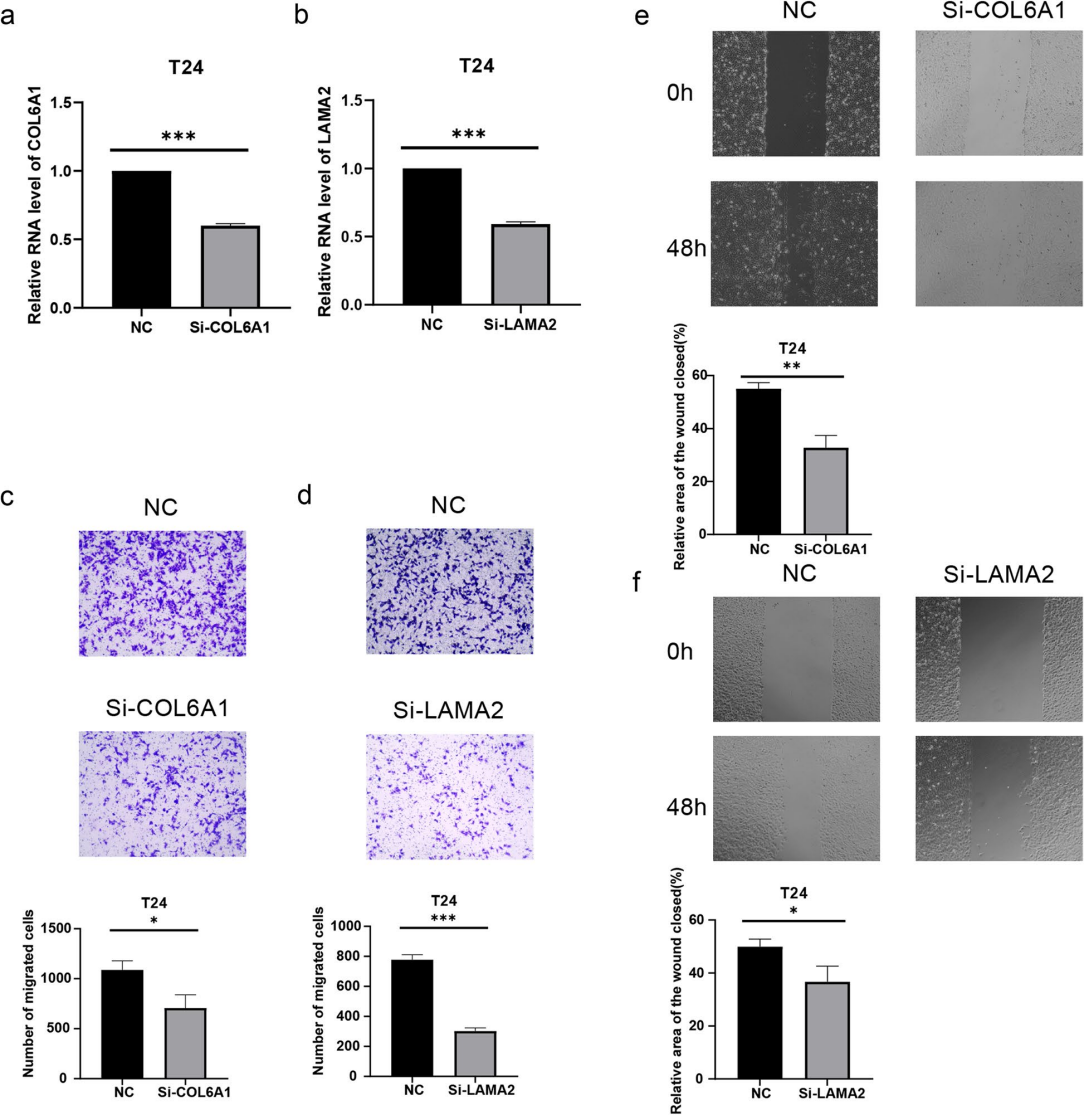
Validation of functional experiments on COL6A1 and LAMA2. **a** Relative mRNA level of COL6A1 after being knocked down. **b** Relative mRNA level of LAMA2 after being knocked down. **c** The number of migrated cells were lower in Si-COL6A1 group than that in NC group. **d** The number of migrated cells was lower in Si-LAMA2 group than that in NC group. **e** Relative wound healing area was lower in Si-COL6A1 group than that in NC group. **f** Relative wound healing area was lower in Si-LAMA2 group than that in NC group

To further investigate the effect of COL6A1 and LAMA2 on the biological function of bladder cancer cells, a series of functional experiments were performed to verify its effect. After knocking down the expression of COL6A1 (Fig. 11a) and LAMA2 (Fig. 11b), the Transwell assay showed that the migration ability of bladder cancer cells was down-regulated (Figs. 11c, d). The result of the wound-healing assay is consistent with that of the Transwell assay (Figs. 11e, f). COL6A1 and LAMA2 were again validated to promote the migration of bladder cancer cells.

## Discussion

Bladder cancer is a common malignancy of the urinary system with unpredictive outcomes. Several bioinformatics studies in recent years have established prognostic models, including an immune genes-related model [23] and an 11-gene model based on 5 cohorts [11], for clinical decision-making. Furthermore, hypoxia-related risk factors [9] and immune landscapes [24] have also been associated with bladder cancer prognosis. In this study, we successfully established a reliable 7-gene focal adhesion-related prognostic model for BLCA using RNA-seq data from the TCGA-BLCA cohort. We verified it on the external GSE32894 dataset. Given the regional differences between the two datasets, the former being from Europe and the latter from North America, we can conclude that the model can be applied universally.

The model comprises of 6 risk genes (VCL, COL6A1, RAC3, PDGFD, JUN, LAMA2) and 1 protective gene (ITGB6), all of which are closely related to focal adhesion. Among the 7 genes, COL6A1 and LAMA2 are the two most significant genes in either KM analysis for predicting prognosis or Kruskal–Wallis rank sum test for combined analysis of expression level and clinical factors, thus, deserve to be further explored.

COL6A1, a gene encoding the collagen VI α1 chain, is widely present in the connective tissues of vertebrates [25]. Collagen VI is a major extracellular matrix protein commonly used to support cell structures. Some studies have shown that collagen VI can regulate cell migration, apoptosis, and tumor progression [26, 27]. Previous studies tend to focus on the function of collagen VI itself, due to its deficiency in myopathy and skeletal muscle diseases [28].

However, COL6A1 has been shown to stimulate proliferation and prevent apoptosis of cancer cells, which has also been found to be related to different types of cancers. For instance, it was proved to be a potential marker of cervical cancer progression in Kazobinka G et al.’s research. The over-expression of COL6A1 was correlated with cervical patients’ prognosis and cell biological functions [29]. Besides, the up-regulation of COL6A1 expression induces tumorigenesis in prostate cancer cells in vivo has also been reported in a study about castration-resistant prostate cancer [30] and enhanced probability of lung cancer cell metastasis in another research [31]. Some researchers also reported that the over-expression of COL6A1 contributed to poor prognosis of renal clear cell carcinoma and glioma patients and enhanced probability of lung cancer cell metastasis [32, 33]. In addition, Snipstad K et al. have reported an up-regulation of extracellular matrix proteins COL6A1 and LAMA4 in rectal cancer after radio‐chemotherapy [34]. All this evidence indicated that COL6A1 has a close relationship with tumor progression and was a novel biomarker of prognosis in different types of cancers, not a simple gene related to collagen anymore.

As a member of the cell adhesion family, LAMA2 also encodes components of extracellular matrix protein called laminin, a glycoprotein in the connective tissue basement membrane, and promotes cell adhesion [35]. Laminin-α2, encoded by LAMA2, is abundant in skeletal muscle, motor nerves, and the brain. It is a composition of trimeric laminin-211 [36] and is an essential constituent of tumor stromal, which can be associated with the malignancy of the tumor. Since damage to the basement membrane of tumor cells plays a vital role in tumor invasion and transfer, many studies were conducted, and evidence has shown that laminin expression was related to tumor progression [37]. LAMA2 belongs to the laminin family. However, seldom researchers paid attention to the direct correlation between LAMA2 overexpression and tumor progression. Most studies focused on LAMA2 deficiency leading to muscular disease, and it is known that mutations in LAMA2 produce a particularly severe type of congenital muscular dystrophy, called LAMA2 chain deficient congenital muscular dystrophy (LAMA2-CMD) [38].

More importantly, seldom studies have explored the relationship between COL6A1, LAMA2, and bladder cancer, thus of great significance in our study.

Besides, other genes in the prognostic model are also related to tumors. For instance, Cheng C et al. claimed RAC3 promoted proliferation, migration, and invasion through PYCR1/JAK/STAT signaling in bladder cancer [39]. VCL has been reported as an important prognostic biomarker in prostate cancer [40]. Satow R et al. reported that PDGFD promotes aggressiveness in prostate and colorectal cancer [41]. Previous research has reported that up-regulation of JUN is associated with the invasiveness of colorectal cancer cells [42]. Singh A et al. reported in 2009 that ITGB6 correlated with a well-differentiated K-Ras-driven cancer such as lung, pancreatic and colon cancer [43].

GSEA of the high- and low-risk groups indicated significant enrichment of biological processes including biosynthesis of unsaturated fatty acids, tight junction, lysine degradation and ubiquitin-mediated proteolysis. proteolysis. Focal adhesion and tight junction commonly belong to enriched cell adhesion/junction pathways [44], indicating that these biological processes can further explore the relationship between tumorigenesis in the future. The risk score also showed more substantial predictive power than multiple clinical factors. Furthermore, patients with advanced clinical features had higher risk scores. Survival analysis indicated that the prognosis of the high-risk group was worse in both the training set and validation set (*p* < 0.01), with respective 3-year AUC values of 0.68 and 0.73, suggesting that the risk score model was capable of predicting BLCA prognosis independent of the clinical factors. The risk score distribution was also similar in both sets, which indicated good consistency and universality of the risk model.

DNA methylation analysis of each 7 genes demonstrated a strong correlation between the gene methylation and overall survival time in the TCGA-BLCA cohort. ITGB6 was a protective factor in our prognostic model and its protective effect was also provn by DNA methylation, as the lower methylation level indicated a better prognosis, which reflected the reliability of the 7-gene prognostic model to some extent. Anuraga G et al. and Wang Z et al. also reported DNA methylation analysis in their gene signature for predicting breast cancer and lung adenocarcinoma via MethSurv [45–47]. The validation of the HPA database on the protein level showed the same tendency as the 7 genes in the prognostic model. Borowczak J et al. reported CDK9 in bladder cancer via the HPA database [48]. The functional experiments successfully verified that COL6A1 promoted the migration of bladder cancer cells as a risk factor, and bladder cancer cells were down-regulated after knocking down the expression of COL6A1. This reflected the reliability of our bioinformatic predicting model.

Previous studies have established tumor-infiltrating lymphocytes as one of the immune-related prognostic factors [49], and CD20 B cell has been identified as a long-term survival factor in BLCA [50]. In this study, we found that both resting and activated DCs were relevant to prognosis. High infiltration of DCs and a high-risk score indicate the worst prognosis. Thus, the infiltration ratio of DCs is a potential new prognostic factor that can be combined with the risk score for more accurate prediction. Furthermore, the correlation between every single gene in the model and Macrophage M2 replenished the evidence that immune cells can influence clinical outcomes, assist in specific immunotherapeutic responses, and help select suitable patients for immunotherapy combined with risk score. Previous studies have reported the relationship between gene and immune cells [51, 52].

However, our results are limited because we only analyzed data from TCGA and GEO databases. Though we validated the model on external cohorts, only the GSE32894 dataset showed an excellent result. GSE13507 and GSE5287 did not establish statistically significant relationships between the expression of each 7genes in the model and survival time on the Prognoscan database. Previous researchers have also drawn from such databases to determine the relationship between genes and prognosis [53, 54] Due to the lack of clinical cohorts, the model’s reliability cannot be verified clinically. And the best cut-off value was directly chosen as a 3-year OS value of ROC without calculating in a more accurate method. The two up-regulated key genes COL6A1 and LAMA2 were not confirmed for their significant roles on the basic experimental level.

## Conclusions

The 7-gene FA-related prognostic model can accurately predict the prognosis of BLCA patients and aid in clinical decision-making. Further studies are needed to amend its accuracy and stability for clinical applications.

## Supplementary Information

**Additional file 1: Supplementary Figure 1.** The ROC curves of (a) risk score, (b) angiolymphatic invasion and (c) age for 1-, 3- and 5-year OS and the corresponding AUC values.

**Additional file 2: Supplementary Figure 2.** Kaplan–Meier survival curves of patients demarcated on the basis of high- and low-DNA methylation of each 7 genes in the model. (a) COL6A1, (b) ITGB6, (c) JUN, (d) LAMA2 (e) PDGFD, (f)RAC3, (g)VCL.

**Additional file 3: Supplementary Figure 3.** Kaplan–Meier survival curves of high- and lowrisk patients demarcated on the basis of (a) activated CD4 memory T cell, (b) dendritic cell, (c) activated mast cells, (d) M1 macrophages and (e) dendritic cell infiltration.

**Additional file 4: Supplementary Figure 4.** Correlation scatter plot of Macrophage M2 infiltration ration and expression level of each 7 genes in the model. (a) COL6A1, (b) ITGB6, (c) JUN, (d) LAMA2, (e) PDGFD, (f) RAC3, (g) VCL.

**Additional file 5: Supplementary Figure 5.** Related validation plots on Prognoscan platform of the RAC3 expression. (a) expression level distribution plot, (b) expression level histogram plot, (c) p-value distribution plot, (d) K-M curves of patients with high- and low-expression of RAC3, (e) survival time distribution plot.

**Additional file 6: Supplementary Figure 6.** Related validation plots on Prognoscan platform of the COL6A1 expression. (a) expression level distribution plot, (b) expression level histogram plot, (c) p-value distribution plot, (d) K-M curves of patients with high- and low-expression of COL6A1, (e) survival time distribution plot.

**Additional file 7: Supplementary Figure 7.** Related validation plots on Prognoscan platform of the ITGB6 expression. (a) expression level distribution plot, (b) expression level histogram plot, (c) p-value distribution plot, (d) K-M curves of patients with high- and low-expression of ITGB6, (e) survival time distribution plot.

**Additional file 8: Supplementary Figure 8.** Validation of the seven-mRNA prognostic signature in the HPA database. The deeper the color, the higher expression in the tissues. (a-b) COL6A1 expression was higher in tumor tissues. (c-d) ITGB6 expression was higher in normal tissues. (e-f) JUN expression was higher in tumor tissues. (g-h) LAMA2 expression differences was insignificant between normal and tumor tissues. (i-j) PDGFD expression was higher in tumor tissues. (k-l) RAC3 expression was higher in tumor tissues. (m-n) VCL expression was higher in tumor tissues.

**Additional file 9: Supplementary Table 1.** Detailed genes table.

**Additional file 10: Supplementary Table 2.** Detailed information of the 7 genes.

**Additional file 11: Supplementary Table 3.** Univariate cox regression analysis of 7 genes (HR: hazard ratio; CI: confidence interval).

**Additional file 12: Supplementary Table 4.** “ESTIMATE” results.

**Additional file 13: Supplementary Table 5.** Sequences of siRNA for transfection.

**Additional file 14: Supplementary Table 6.** Sequences of primer pair for qPCR.

## Data Availability

The study’s GEO dataset (GSE32894) can be downloaded from https://www.ncbi.nlm.nih.gov/gds/. TCGA-BLCA gene matrix (HTSeq-FPKM) and clinical data can be obtained from https://xenabrowser.net and http://www.cbioportal.org.

## Abbreviations

BLCA: Bladder cancer
TCGA: The Cancer Genome Atlas
GEO: Gene Expression Omnibus
KEGG: Kyoto Encyclopedia of Genes and Genomes
LASSO: Least absolute shrinkage and selection operator
GSEA: Gene set enrichment analysis
ROC: Receiver operating characteristic
KM: Kaplan–Meier survival analysis
NMIBC: Non-muscular invasive bladder cancer
MIBC: Muscular invasive bladder cancer
TRUBT: Transurethral resection of bladder tumors
FDA: Food and Drug Administration
FA: Focal adhesion
ECM: Extracellular matrix
EMT: Epithelial–mesenchymal transition
DEGs: Differentially expressed genes
MSigDB: Molecular Signatures Database
siRNA: Small interfering RNA
qRT-PCR: Quantitative real-time PCR
AUC: Area under the curve
OS: Overall survival
LR: Likelihood ratio
HPA: Human Protein Atlas
DCs: Dendritic cells
LAMA2-CMD: LAMA2 chain deficient congenital muscular dystrophy
