# Supplementary material for: A novel focal adhesion-related risk model predicts prognosis of bladder cancer —— a bioinformatic study based on TCGA and GEO database
Source: BMC Cancer. 2022 Nov 10;22:1158. doi: 10.1186/s12885-022-10264-5 (PMC9647995; doi:10.1186/s12885-022-10264-5)
Supplement: Supplementary file 1 — Additional file 1: Supplementary Figure 1. The ROC curves of (a) risk score, (b) angiolymphatic invasion and (c) age for 1-, 3- and 5-year OS and the corresponding AUC values. [file 12885_2022_10264_MOESM1_ESM.pdf]

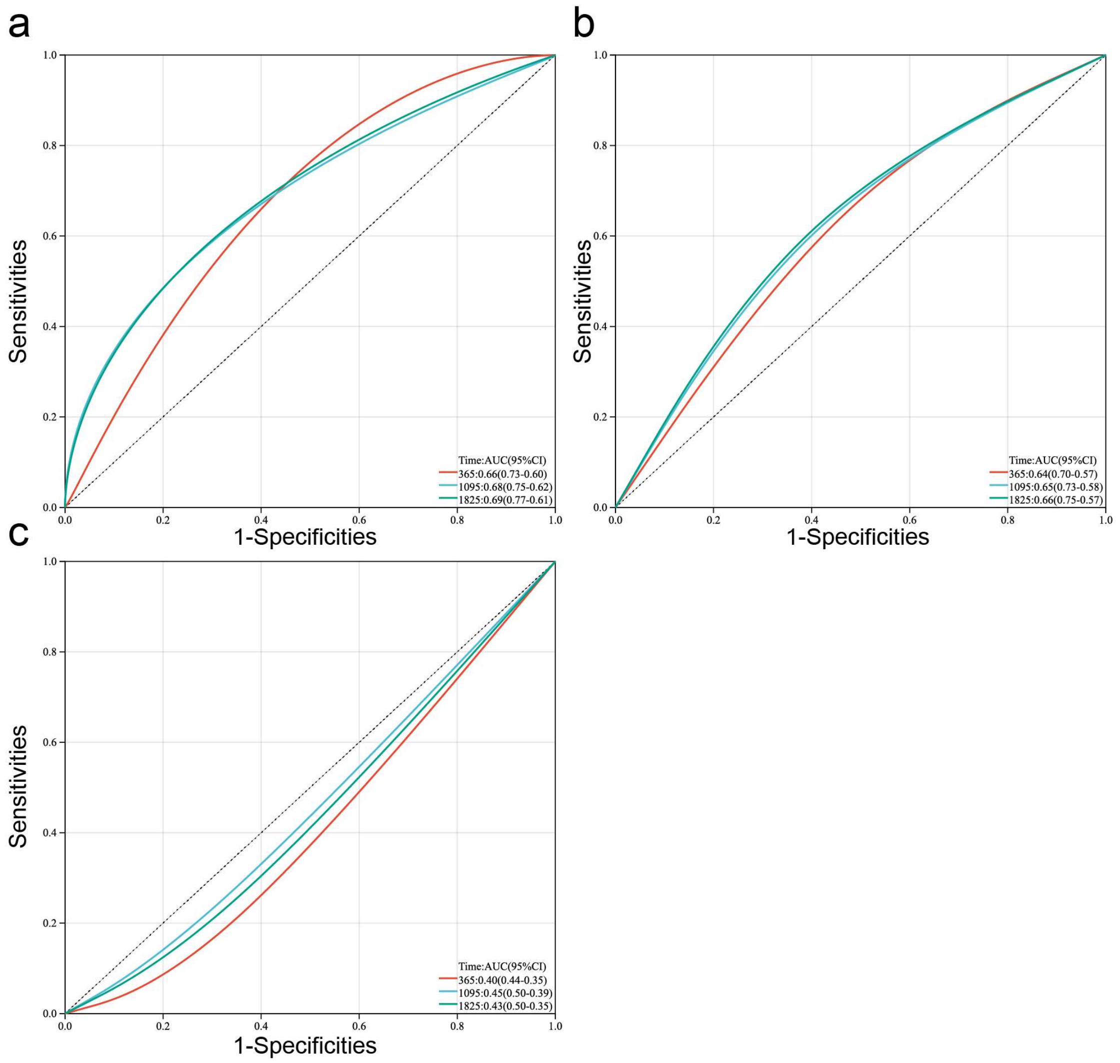

Supplementary Figure 1. The ROC curves of (a) risk score, (b) angiolymphatic invasion and (c) age for 1-, 3- and 5-year OS and the corresponding AUC values.
