# Supplementary material for: A novel focal adhesion-related risk model predicts prognosis of bladder cancer —— a bioinformatic study based on TCGA and GEO database
Source: BMC Cancer. 2022 Nov 10;22:1158. doi: 10.1186/s12885-022-10264-5 (PMC9647995; doi:10.1186/s12885-022-10264-5)
Supplement: Supplementary file 2 — Additional file 2: Supplementary Figure 2. Kaplan–Meier survival curves of patients demarcated on the basis of high- and low-DNA methylation of each 7 genes in the model. (a) COL6A1, (b) ITGB6, (c) JUN, (d) LAMA2 (e) PDGFD, (f)RAC3, (g)VCL. [file 12885_2022_10264_MOESM2_ESM.pdf]

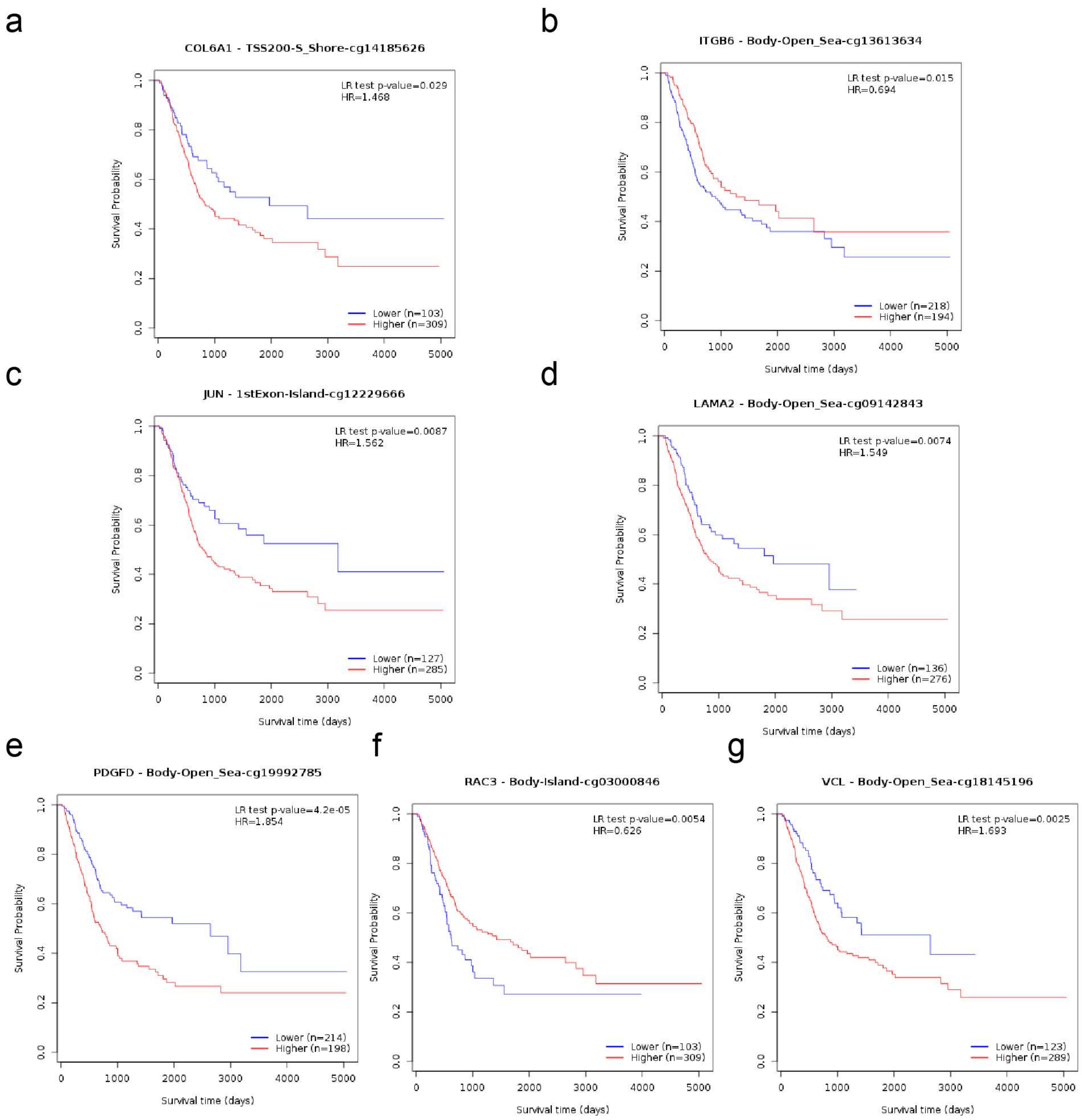

Supplementary Figure 2. Kaplan–Meier survival curves of patients demarcated on the basis of high- and low-DNA methylation of each 7 genes in the model. (a) COL6A1, (b) ITGB6, (c) JUN, (d) LAMA2 (e) PDGFD, (f)RAC3, (g)VCL.
