# Supplementary material for: A novel focal adhesion-related risk model predicts prognosis of bladder cancer —— a bioinformatic study based on TCGA and GEO database
Source: BMC Cancer. 2022 Nov 10;22:1158. doi: 10.1186/s12885-022-10264-5 (PMC9647995; doi:10.1186/s12885-022-10264-5)
Supplement: Supplementary file 3 — Additional file 3: Supplementary Figure 3. Kaplan–Meier survival curves of high- and lowrisk patients demarcated on the basis of (a) activated CD4 memory T cell, (b) dendritic cell, (c) activated mast cells, (d) M1 macrophages and (e) dendritic cell infiltration. [file 12885_2022_10264_MOESM3_ESM.pdf]

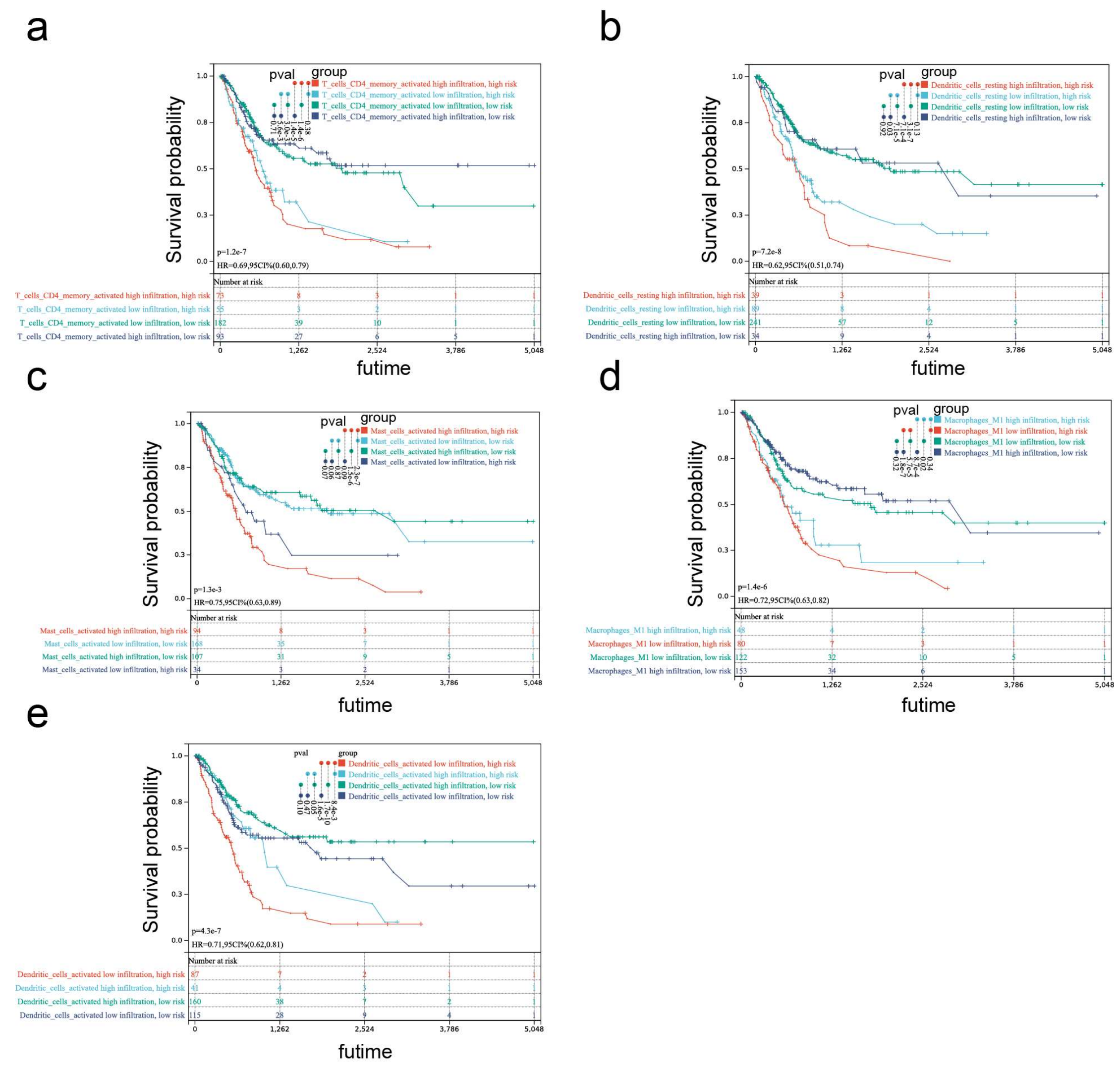

Supplementary Figure 3. Kaplan–Meier survival curves of high- and low-risk patients demarcated on the basis of (a) activated CD4 memory T cell, (b) dendritic cell, (c) activated mast cells, (d) M1 macrophages and (e) dendritic cell infiltration.
