# Supplementary material for: A novel focal adhesion-related risk model predicts prognosis of bladder cancer —— a bioinformatic study based on TCGA and GEO database
Source: BMC Cancer. 2022 Nov 10;22:1158. doi: 10.1186/s12885-022-10264-5 (PMC9647995; doi:10.1186/s12885-022-10264-5)
Supplement: Supplementary file 4 — Additional file 4: Supplementary Figure 4. Correlation scatter plot of Macrophage M2 infiltration ration and expression level of each 7 genes in the model. (a) COL6A1, (b) ITGB6, (c) JUN, (d) LAMA2, (e) PDGFD, (f) RAC3, (g) VCL. [file 12885_2022_10264_MOESM4_ESM.pdf]

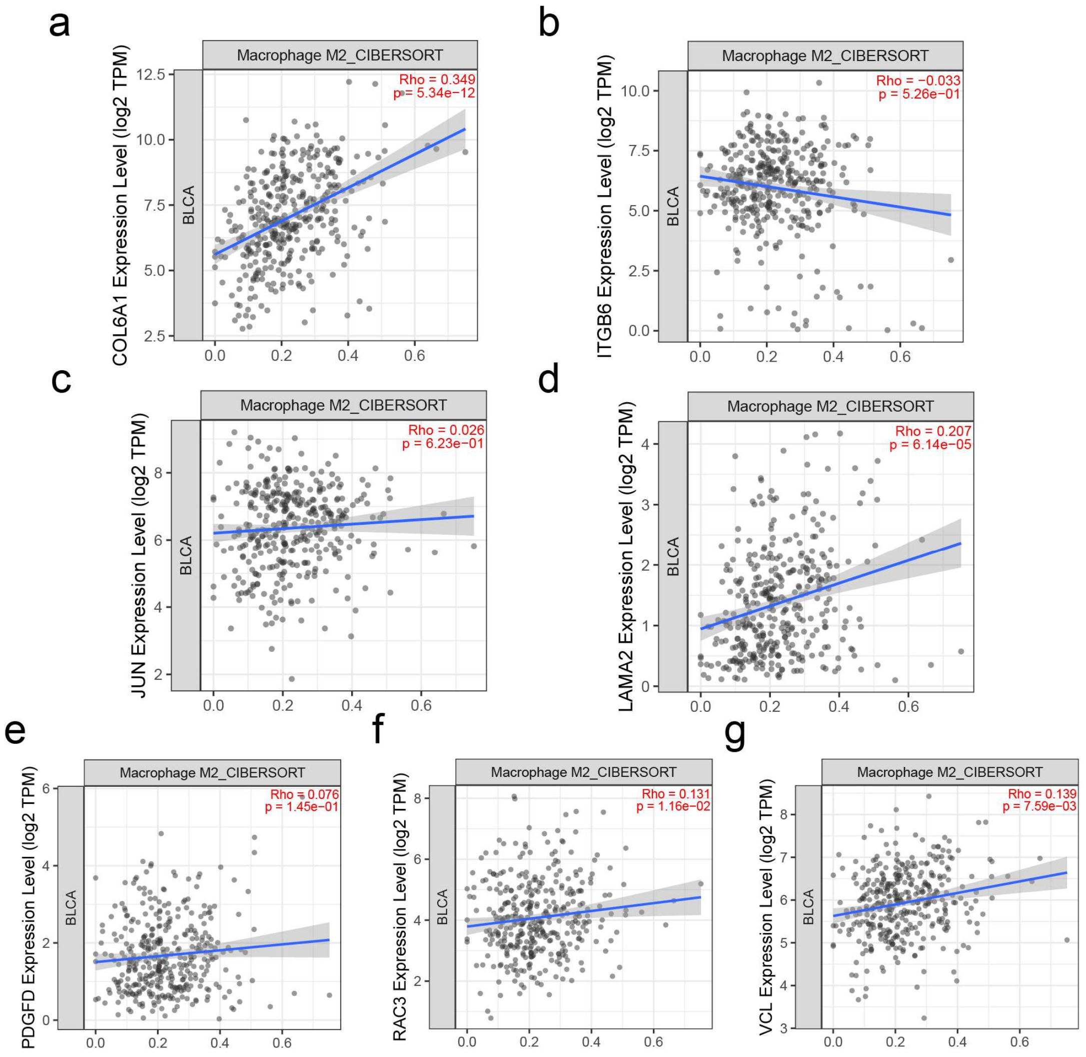

Supplementary Figure 4. Correlation scatter plot of Macrophage M2 infiltration ration and expression level of each 7 genes in the model. (a) COL6A1, (b) ITGB6, (c) JUN, (d) LAMA2, (e) PDGFD, (f) RAC3, (g) VCL.
