# Supplementary material for: A novel focal adhesion-related risk model predicts prognosis of bladder cancer —— a bioinformatic study based on TCGA and GEO database
Source: BMC Cancer. 2022 Nov 10;22:1158. doi: 10.1186/s12885-022-10264-5 (PMC9647995; doi:10.1186/s12885-022-10264-5)
Supplement: Supplementary file 6 — Additional file 6: Supplementary Figure 6. Related validation plots on Prognoscan platform of the COL6A1 expression. (a) expression level distribution plot, (b) expression level histogram plot, (c) p-value distribution plot, (d) K-M curves of patients with high- and low-expression of COL6A1, (e) survival time distribution plot. [file 12885_2022_10264_MOESM6_ESM.pdf]

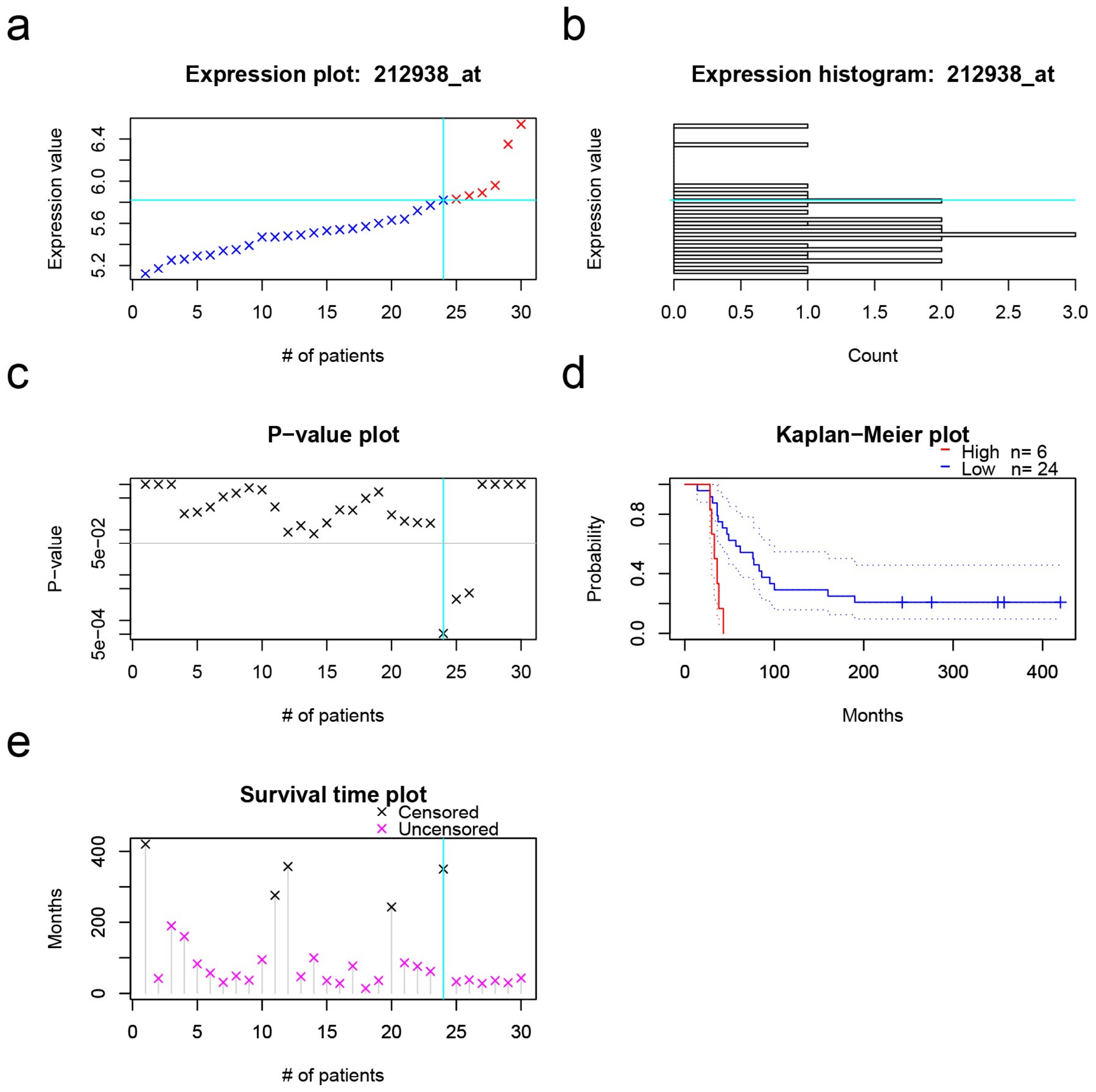

Supplementary Figure 6. Related validation plots on Prognoscan platform of the COL6A1 expression. (a) expression level distribution plot, (b) expression level histogram plot, (c) *p*-value distribution plot, (d) K-M curves of patients with high- and low-expression of COL6A1, (e) survival time distribution plot.
