# Supplementary material for: A novel focal adhesion-related risk model predicts prognosis of bladder cancer —— a bioinformatic study based on TCGA and GEO database
Source: BMC Cancer. 2022 Nov 10;22:1158. doi: 10.1186/s12885-022-10264-5 (PMC9647995; doi:10.1186/s12885-022-10264-5)
Supplement: Supplementary file 8 — Additional file 8: Supplementary Figure 8. Validation of the seven-mRNA prognostic signature in the HPA database. The deeper the color, the higher expression in the tissues. (a-b) COL6A1 expression was higher in tumor tissues. (c-d) ITGB6 expression was higher in normal tissues. (e-f) JUN expression was higher in tumor tissues. (g-h) LAMA2 expression differences was insignificant between normal and tumor tissues. (i-j) PDGFD expression was higher in tumor tissues. (k-l) RAC3 expression was higher in tumor tissues. (m-n) VCL expression was higher in tumor tissues. [file 12885_2022_10264_MOESM8_ESM.pdf]

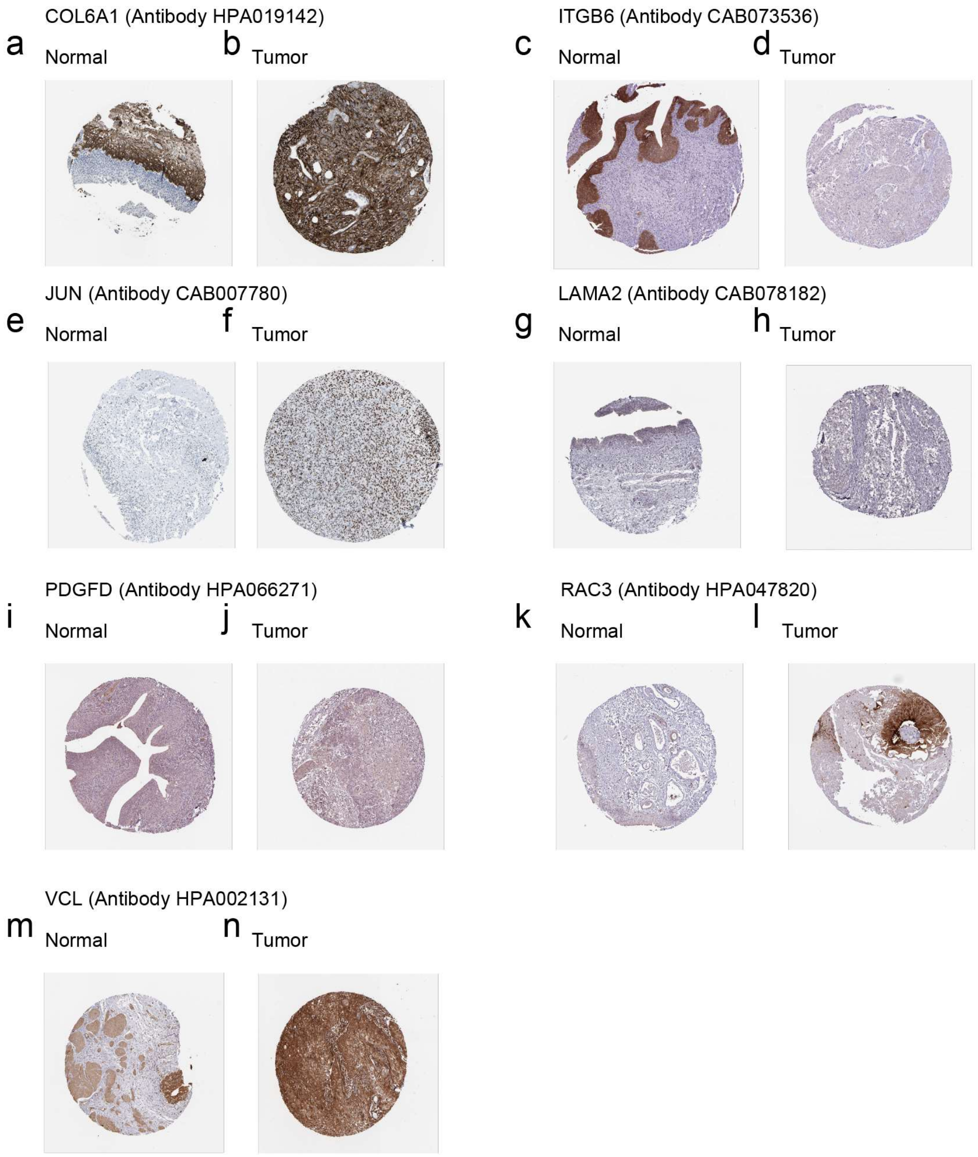

Supplementary Figure 8. Validation of the seven-mRNA prognostic signature in the HPA database. The deeper the color, the higher expression in the tissues. (a-b) COL6A1 expression was higher in tumor tissues. (c-d) ITGB6 expression was higher in normal tissues. (e-f) JUN expression was higher in tumor tissues. (g-h) LAMA2 expression differences was insignificant between normal and tumor tissues. (i-j) PDGFD expression was higher in tumor tissues. (k-l) RAC3 expression was higher in tumor tissues. (m-n) VCL expression was higher in tumor tissues.
