# Supplementary material for: A novel focal adhesion-related risk model predicts prognosis of bladder cancer —— a bioinformatic study based on TCGA and GEO database
Source: BMC Cancer. 2022 Nov 10;22:1158. doi: 10.1186/s12885-022-10264-5 (PMC9647995; doi:10.1186/s12885-022-10264-5)
Supplement: Supplementary file 10 — Additional file 10: Supplementary Table 2. Detailed information of the 7 genes. [file 12885_2022_10264_MOESM10_ESM.pdf]

Supplementary Table 2: Detailed information of the 7 genes				
**Gene Ensemble ID**	**Gene type**	**Gene name**	**Location**	**Coef**
ENSG00000035403	Protein coding	VCL	Chromosome 10: 73,995,193-74,121,363	0.14523168
ENSG00000115221	Protein coding	ITGB6	Chromosome 2: 160,099,667-160,200,313	-0.0832307
ENSG00000142156	Protein coding	COL6A1	Chromosome 21: 45,981,770-46,005,050	0.00766304
ENSG00000169750	Protein coding	RAC3	Chromosome 17: 82,031,678-82,034,204	0.24038421
ENSG00000170962	Protein coding	PDGFD	Chromosome 11: 103,907,189-104,164,379	0.08168648
ENSG00000177606	Protein coding	JUN	Chromosome 1: 58,776,845-58,784,048	0.11920731
ENSG00000196569	Protein coding	LAMA2	Chromosome 6: 128,883,138-129,516,566	0.19265823
