# Supplementary material for: A novel focal adhesion-related risk model predicts prognosis of bladder cancer —— a bioinformatic study based on TCGA and GEO database
Source: BMC Cancer. 2022 Nov 10;22:1158. doi: 10.1186/s12885-022-10264-5 (PMC9647995; doi:10.1186/s12885-022-10264-5)
Supplement: Supplementary file 11 — Additional file 11: Supplementary Table 3. Univariate cox regression analysis of 7 genes (HR: hazard ratio; CI: confidence interval). [file 12885_2022_10264_MOESM11_ESM.pdf]

Supplementary Table 3: Univariate cox regression analysis of 7 genes (HR: hazard ratio; CI: confidence interval)

Gene	TCGA-BLCA	
	HR (95%CI)	P
VCL	1.30(1.08-1.57)	5.14E-03
ITGB6	0.87(0.79-0.97)	7.70E-03
COL6A1	1.16(1.07-1.27)	6.35E-04
RAC3	1.20(1.05-1.39)	9.90E-03
PDGFD	1.28(1.09-1.52)	3.47E-03
JUN	1.19(1.05-1.35)	6.33E-03
LAMA2	1.41(1.19-1.66)	5.13E-05
