# Supplementary material for: A novel focal adhesion-related risk model predicts prognosis of bladder cancer —— a bioinformatic study based on TCGA and GEO database
Source: BMC Cancer. 2022 Nov 10;22:1158. doi: 10.1186/s12885-022-10264-5 (PMC9647995; doi:10.1186/s12885-022-10264-5)
Supplement: Supplementary file 13 — Additional file 13: Supplementary Table 5. Sequences of siRNA for transfection. [file 12885_2022_10264_MOESM13_ESM.pdf]

Supplementary Table 5: Sequences of siRNA for transfection

Gene	Nucleotide sequence(5'-3')
Si-COL6A1	ACAUUUUCAUGAUGAUGUCCA
	GACAUCAUCAUGAAAAUGUGC
Si-LAMA2	F:TGCTGTCCTGAATCTTGCTTC
	R:AGCATTTGTAATCGGGTGTCTC
