# Supplementary material for: A novel focal adhesion-related risk model predicts prognosis of bladder cancer —— a bioinformatic study based on TCGA and GEO database
Source: BMC Cancer. 2022 Nov 10;22:1158. doi: 10.1186/s12885-022-10264-5 (PMC9647995; doi:10.1186/s12885-022-10264-5)
Supplement: Supplementary file 14 — Additional file 14: Supplementary Table 6. Sequences of primer pair for qPCR. [file 12885_2022_10264_MOESM14_ESM.pdf]

Supplementary Table 6: Sequences of primer pair for qPCR

Gene	Nucleotide sequence(5'-3')
COL6A1	F:ACAGTGACGAGGTGGAGATCA
	R:GATAGCGCAGTCGGTGTAGG
LAMA2	F:TGCTGTCCTGAATCTTGCTTC
	R:AGCATTTGTAATCGGGTGTCTC
GAPDH	F:GGAGCGAGATCCCTCCAAAAT
	R:GGCTGTTGTCATACTTCTCATGG

F, forward; R, reverse
